# Macrophage GLUD1 is Essential for Redox Balance and Immune Homeostasis in Cardiac Injury

**DOI:** 10.1002/advs.78416

**Published:** 2026-10-28

**Authors:** Tingting Jin, Chaojie Lai, Qingyan Zhou, Ruilin Wang, Yiping Tong, Wentao Chen, Nikola Rabrenovic, Tingyu Zhang, Xinrong Ren, Chengchen Huang, Jiawen Chen, Ran Li, Luyang Yu, Massimiliano Mazzone, Wenbin Zhang, Guosheng Fu, Min Shang

**Affiliations:** ^1^ Zhejiang Key Laboratory of Cardiovascular Intervention and Precision Medicine Engineering Research Center For Cardiovascular Innovative Devices of Zhejiang Province Department of Cardiology Sir Run Run Shaw Hospital School of Medicine Zhejiang University Hangzhou China; ^2^ School of Basic Medical Sciences and Forensic Medicine Hangzhou Medical College Hangzhou Zhejiang China; ^3^ Zhejiang Key Laboratory of Cardiovascular Intervention and Precision Medicine of Sir Run Run Shaw Hospital Zhejiang University‐Lishui Joint Innovation Center For Life and Health Zhejiang University Hangzhou China; ^4^ College of Life Sciences‐iCell Biotechnology Regenerative Biomedicine Laboratory College of Life Sciences Zhejiang University Hangzhou China; ^5^ Laboratory of Tumor Inflammation and Angiogenesis Center For Cancer Biology VIB Leuven Belgium; ^6^ Laboratory of Tumor Inflammation and Angiogenesis Center For Cancer Biology Department of Oncology KU Leuven Leuven Belgium

**Keywords:** acute myocardial infarction, cardiac Injury, cell metabolism, glutamate dehydrogenase 1 (GLUD1), immune homeostasis, immunometabolism, ischemia‐reperfusion injury, monocytes and macrophages, reactive oxygen species

## Abstract

Excessive free radical production and dysregulated immune responses contribute to myocardial ischemia‐reperfusion (IR) injury. However, the mechanisms by which macrophages regulate reactive oxygen species (ROS) homeostasis and orchestrate immune responses during IR injury remain elusive. Here, we show that glutamate dehydrogenase 1 (GLUD1) expression is induced in monocytes from patients with acute myocardial infarction after interventional therapy and in cardiac‐recruited macrophages from mice subjected to IR injury. GLUD1‐deficient macrophages exacerbate IR injury by increasing mitochondrial ROS accumulation and amplifying pro‐inflammatory responses. Mechanistically, GLUD1 deficiency leads to mitochondrial ROS accumulation due to reduced NADPH/NADP^+^ and GSH/GSSG ratios during IR injury. Elevated mitochondrial ROS inhibits PHD2 activity, stabilizing HIF‐1α and enhancing NF‐κB signaling, promoting the release of pro‐inflammatory factors and exacerbating myocardial damage. Systemic activation of GLUD1 or adoptive transfer of monocytes pre‐treated with a GLUD1 activator preserved cardiac function and mitigated IR injury. These findings highlight macrophage GLUD1 as a critical regulator of redox balance and immune homeostasis, presenting a promising therapeutic target for alleviating IR injury and preserving cardiac function.

## Graphical abstract

Excessive free radical production and dysregulated immune responses are key contributors to myocardial ischemia‐reperfusion (IR) injury. Macrophage GLUD1 activity safeguards against HIF‐1α accumulation and maladaptive responses to IR‐induced ROS production. Specifically, GLUD1 deficiency results in mitochondrial ROS accumulation due to reduced NADPH/NADP^+^ and GSH/GSSG ratios during IR injury. Elevated mitochondrial ROS inhibits PHD2 activity, leading to HIF‐1α accumulation, while promoting IκBα degradation and NF‐κB activation. This cascade triggers the release of pro‐inflammatory cytokines, ultimately causing cardiomyocyte apoptosis and cell death during IR. Therapeutically, pharmacological activation of GLUD1 preserves cardiac function and mitigates IR injury. Figure created in BioRender, Shang, M. (2026) ntt54v1.

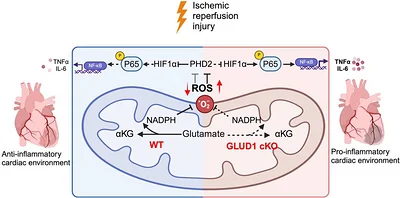

## 1 Introduction

Acute myocardial infarction (AMI) is one of the most dangerous cardiovascular diseases in clinical practice, characterized by high rates of mortality and disability [1, 2]. It poses a significant threat to human health and burdens society and the economy considerably [3, 4]. Timely and effective reperfusion therapy is crucial for salvaging cardiomyocytes at risk due to ischemia. However, the benefits of reperfusion are often undermined by myocardial ischemia‐reperfusion (IR) injury, which exacerbates cardiomyocyte damage and limits therapeutic efficacy [5]. Clinically, IR injury manifests as adverse events such as sudden hypotension, malignant arrhythmias, heart failure, and, in severe cases, sudden cardiac death. Currently, there are few established clinical solutions for mitigating these complications [6, 7].

IR injury is a multifaceted pathological process involving metabolic alterations, inflammation, oxidative stress, and microvascular obstruction [6, 8, 9]. Among these, inflammation and the overproduction of reactive oxygen species (ROS) play pivotal roles [10]. ROS accumulation is a major driver of early tissue damage during ischemia and reperfusion [11, 12]. However, few studies have addressed the role of macrophage ROS scavenging in IR injury [13, 14]. Macrophages and monocytes constitute the most abundant and diverse cell populations among cardiac leukocytes following IR injury [15]. Emerging evidence highlights the critical roles of macrophages in IR injury, including their involvement in infiltration, immune response modulation, debris clearance, cardiac remodeling, and the resolution of inflammation [13, 16, 17, 18]. Persistent inflammatory activation of macrophages, however, exacerbates IR injury. Despite these advances, how macrophages restore cardiac immune balance and redox homeostasis remains incompletely understood.

Glutaminolysis, a key metabolic pathway fueling the immune responses, has been strongly associated with the risk of cardiovascular diseases, as demonstrated in several case‐cohort studies [19, 20, 21, 22, 23]. Glutamate dehydrogenase 1 (GLUD1, also known as GDH) is a mitochondrial enzyme ubiquitous in living organisms, playing a pivotal role in nitrogen and glutamate metabolism, as well as energy homeostasis. While the unique form of GLUD1 found in mice is universally expressed in humans, GLUD2 is expressed explicitly in testicular tissue and astrocytes. GLUD1 catalyzes the conversion of glutamate to α‐ketoglutaric acid, accompanied by the reduction of NADP^+^ to NADPH or NAD^+^ to NADH [24]. The ratios of NADPH/NADP^+^ or NADH/NAD^+^ affect the cellular redox balance [25]. We have previously shown that the deficiency of GLUD1 in macrophages nourishes the pool of satellite cells and promotes their differentiation by increasing macrophage‐derived glutamine production and secretion [26]. Here, we questioned whether and how macrophage GLUD1 orchestrates cardiac redox and immune homeostasis.

In this study, we identified a significant upregulation of GLUD1 in monocyte‐derived macrophages during myocardial IR injury by single‐cell analysis. Additionally, we examined the clinical and pathological significance of macrophage GLUD1 in IR injury by assessing its induced expression in circulating monocytes from patients with acute myocardial infarction undergoing interventional therapy. Our findings revealed that GLUD1 deficiency in macrophages exacerbates myocardial IR injury by promoting a pro‐inflammatory phenotype and increasing ROS levels. Mechanistically, GLUD1 knockout macrophages reduced NADPH/NADP^+^ and GSH/GSSG ratios, leading to an elevation in mitochondrial ROS levels. This ROS accumulation inhibited prolyl hydroxylase domain protein 2 (PHD2) activity, subsequently stabilizing hypoxia‐inducible factor‐1α (HIF‐1α) and activating nuclear factor‐κB (NF‐κB), thus triggering pro‐inflammatory responses in macrophages. From a therapeutic perspective, we demonstrated that systemic administration of the GLUD1 activator BCH, as well as transfusion of BCH‐treated monocytes, effectively preserved cardiac function and reduced myocardial injury during IR, paving the way for alternative treatment strategies for ischemic heart diseases.

## 2 Results

### 2.1 *Glud1* Expression is Upregulated in Monocytes/Macrophages During Myocardial Ischemia‐Reperfusion Injury

To investigate the association between macrophage GLUD1 expression and cardiac ischemia‐reperfusion (IR) injury, we evaluated *Glud1* expression in myocardial macrophages after IR injury using single‐cell RNA sequencing analysis. Analysis of the public dataset GSE247139 identified seven monocyte/macrophage subpopulations (Figure 1a,b, Figure S1a,b), among which Ly6C^+^ monocytes exhibited the highest *Glud1* expression (Figure 1c). Pseudotime trajectory analysis was then performed to characterize the dynamics of *Glud1* expression during monocyte‐to‐macrophage differentiation. This analysis revealed that the trajectory originated from Ly6C^+^ monocytes and branched into three terminal subsets: MHC‐II^+^ macrophages, Isg^+^ macrophages, and Mt^hi^ macrophages. The Blt1^+^ and S100a9^+^ subpopulations, which were markedly expanded after IR injury, occupied obligatory intermediate states along these differentiation trajectories (Figure 1b, Figure S1c). Consistently, the inflammatory score peaked in the Blt1^+^ and S100a9^+^ intermediate populations and subsequently declined in the terminal subsets (Figure S1d,e), indicating a transient pro‐inflammatory intermediate state during macrophage differentiation. Gene Ontology (GO) analyses of the Blt1^+^ and S100a9^+^ populations further highlighted the involvement of macrophage immune responses during IR injury (Figure S1f,g). Notably, *Glud1* expression was highest in Ly6C^+^ monocytes, which were positioned at the origin of the trajectory and preceded the pro‐inflammatory Blt1^+^ and S100a9^+^ intermediate stages (Figure 1c, Figure S1c–e). This temporal pattern suggests that *Glud1* is enriched in recruited monocytes before the peak of inflammatory macrophage activation, indicating that *Glud1* upregulation is an early feature of the recruited macrophage response to myocardial IR injury.

**FIGURE 1:**
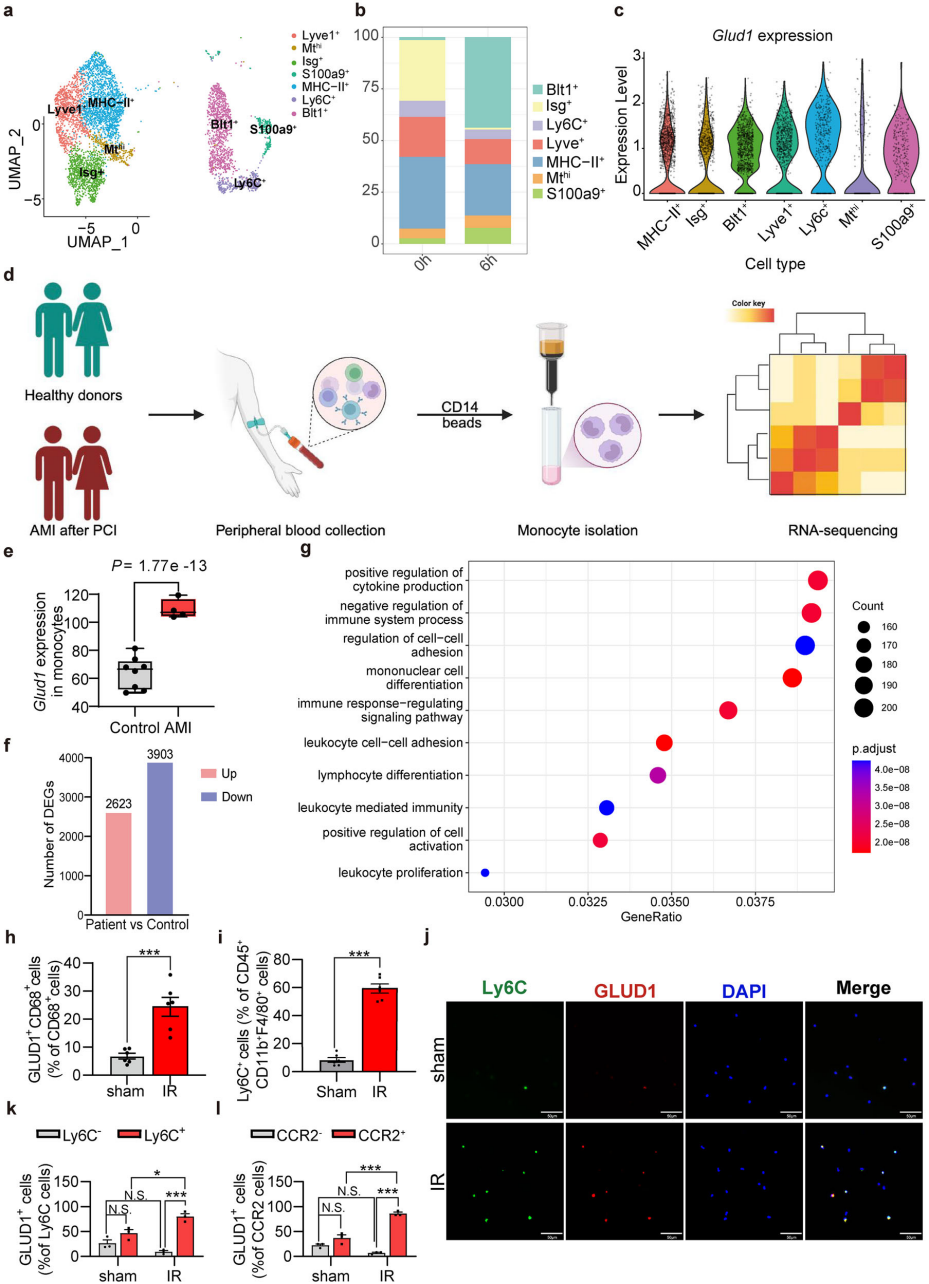
*Glud1* expression is upregulated in monocytes/macrophages during myocardial ischemia‐reperfusion injury. (a) Clustering of cardiac monocyte/macrophage populations from mice subjected to sham‐operated or myocardial ischemia‐reperfusion (IR) injury. Seven major monocyte/macrophage clusters were identified. (b) Bar plot displaying the percentage of each monocyte/macrophage subset. (c) Violin plot showing *Glud1* expression across monocyte/macrophage subsets. (d) Scheme illustrating the strategy for isolating peripheral blood monocytes from acute myocardial infarction (AMI) patients post‐intervention and healthy donors. Figure created in BioRender, Shang, M. (2026) x6g3sc8. (e) *GLUD1* expression in peripheral blood monocytes from AMI patients post intervention vs. healthy donors (healthy donors,n=8; AMI patients, n=4). (f) Number of differentially expressed genes (DEGs) in peripheral blood monocytes from AMI patients compared to healthy donors, with red and blue bars indicating significantly upregulated and downregulated genes, respectively. (g) Top 10 enriched biological processes based on GO analysis of DEGs in peripheral blood monocytes from patients and healthy donors. (h) Quantification of CD68^+^ cells and GLUD1 expression in CD68^+^ cells from sham operated and IR mice (n=6 per group). (i) Flow cytometric analysis of Ly6C^+^ cells within the cardiac CD45^+^CD11b^+^F4/80^+^ monocyte/macrophage population from WT mice 24 h after sham operation and IR injury (n=6 per group). (j‐k) Representative images and quantification of Ly6C and GLUD1 expression in cardiac CD45^+^CD11b^+^F4/80^+^ cells isolated from WT mice subjected to sham operation and IR injury. The percentages of GLUD1^+^ cells within the Ly6C^+^ and Ly6C^−^ subsets were quantified (n=3). Scale bar, 50 µm. (l) Quantification of the percentages of GLUD1^+^ cells within the CCR2^+^ and CCR2^−^ subsets (n=3). Graphs show mean ± SEM. All experiments were independently replicated in triplicate. Statistical analyses were conducted using a two‐tailed unpaired t‐test for e,h,i and two way ANOVA followed by Tukey's test for k,l. ^*^ *P* < 0.05, ^***^ *P* < 0.0005, N.S., not significant (*P* > 0.05).

To validate these observations in human samples, peripheral blood monocytes were isolated from patients with acute myocardial infarction (AMI) after interventional therapy and from healthy donors using CD14^+^ beads. Bulk RNA‐sequencing analysis revealed significantly elevated *GLUD1* expression in monocytes from AMI patients compared to healthy controls (Figure 1d,e). Gene Ontology (GO) and KEGG pathway analyses of these monocytes highlighted the involvement of macrophage immune responses during IR injury (Figure 1f,g, Figure S1h). Consistent with these findings, in a mouse IR injury model, with 45 min of induced ischemia followed by 24 h of reperfusion, as previously described [13, 18, 27], we observed increased infiltration of CD68^+^ monocytes/macrophages into cardiac tissue post‐IR injury (Figure S2a,b). Notably, GLUD1 expression in cardiac monocytes/macrophages was elevated approximately threefold compared with sham controls (Figure 1 h). In parallel, the Ly6C^+^ recruited cardiac monocyte/macrophage population was significantly expanded after IR injury compared with sham operation (Figure 1i, Figure S2c). Immunofluorescence staining of sorted cardiac CD45^+^CD11b^+^F4/80^+^ monocytes/macrophages showed higher GLUD1 expression in Ly6C^+^ and CCR2^+^ cells than in their negative counterparts after myocardial IR injury. Within these subsets, GLUD1 expression was further increased after IR injury compared with sham operation (Figure 1j–l, Figure S2d). Overall, these data indicate an upregulation of *Glud1* expression in monocyte‐derived cardiac macrophages in response to myocardial ischemia‐reperfusion injury.

### 2.2 Macrophage GLUD1 Deficiency Exacerbates Cardiac Ischemia‐Reperfusion Injury

To elucidate the role of macrophage *Glud1* in IR injury, we employed myeloid‐specific *Glud1* knockout mice (*Glud1* ^L/L^ x LysM: Cre) and inducible monocyte/macrophage *Glud1* knockout mice (*Glud1* ^L/L^ x CSF1R: Cre‐ERT), from here on referred to as *Glud1* ^ΔMo^ and *Glud1* ^iΔMac^, respectively [26]. BMDMs isolated from both *Glud1* ^ΔMo^ and *Glud1* ^iΔMac^ mice exhibited significantly reduced GLUD1 expression compared to wild‐type (WT) littermate controls (Figure S3a,b), confirming the efficient and specific deletion of *Glud1* in macrophages in these two strains. To assess the impact of *Glud1* deletion in macrophages on IR injury, we first evaluated infarct size using Evan's Blue and TTC double staining. *Glud1* ^ΔMo^ mice exhibited significantly larger infarct sizes post‐IR injury compared to WT controls (Figure 2a,b). There was no significant difference in the ischemic risk area between the WT and *Glud1* ^ΔMo^ mice, indicating that the extent of myocardial infarction due to IR injury was similar (Figure 2c). To further characterize the impact of *Glud1* deletion on myocardial apoptosis, we performed TUNEL staining to quantify apoptotic cells in the myocardium. *Glud1* ^ΔMo^ mice exhibited a significantly higher incidence of apoptosis post‐IR injury, as evidenced by an increased number of TUNEL^+^ cells compared to WT controls (Figure 2d,e). In addition to increased infarct size and apoptosis, echocardiographic analysis revealed impaired cardiac contractile function in *Glud1* ^ΔMo^ mice. Post‐IR injury, *Glud1* ^ΔMo^ mice displayed significantly reduced ejection fraction (EF) and fractional shortening (FS) compared to WT controls (Figure 2f,g, Table S1). To further validate these observations, we utilized the inducible *Glud1* ^iΔMac^ mouse model. Consistent with the results observed in *Glud1* ^ΔMo^ mice, inducible deletion of *Glud1* in *Glud1* ^iΔMac^ mice resulted in significantly larger infarct sizes compared to WT controls (Figure 2h‐j). This further validates the detrimental impact of GLUD1 loss in macrophages on myocardial injury and cardiac functions.

**FIGURE 2:**
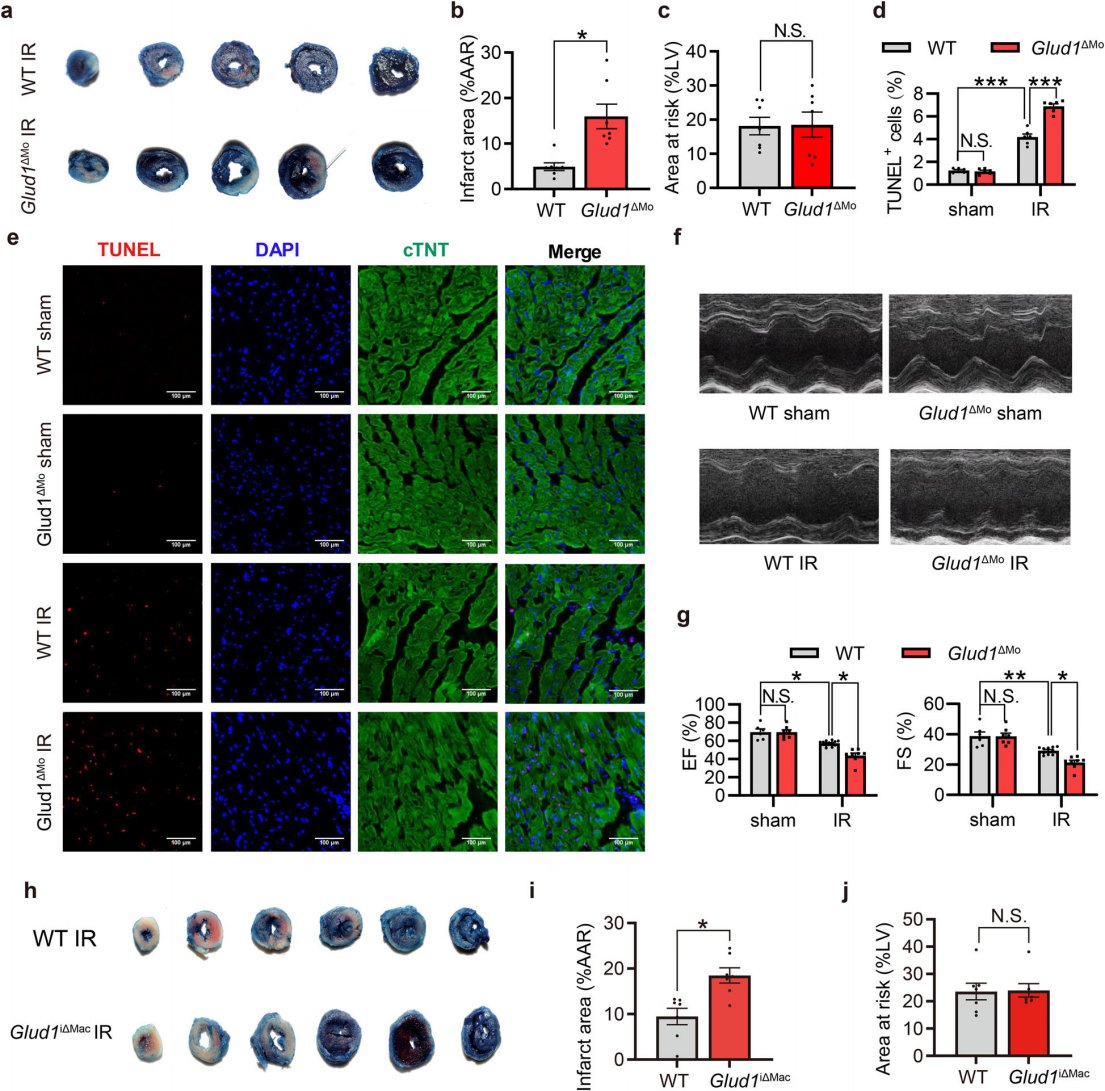
Macrophage GLUD1 deficiency exacerbates cardiac ischemia‐reperfusion injury. (a‐c) Left ventricular (LV) tissue sections from WT and *Glud1* ^ΔMo^ mice stained with Evans Blue and TTC to evaluate the myocardial infarction area at 24 h post‐IR. The area at risk (AAR, ischemic and border) and the relative ratio of the infarct region (IF) to the AAR were quantified (n=7 per group). (d‐e) Quantification and representative images of TUNEL‐positive cells in hearts of WT and *Glud1* ^ΔMo^ post‐myocardial IR (n=6 per group). Scale bar, 100 µm. (f‐g) Representative echocardiography images and quantification of ejection fraction (EF) and fractional shortening (FS) in WT and *Glud1^ΔMo^* mice after sham operation (WT, n=6; GLUD1 cKO, n=7) or IR injury (WT, n=10; GLUD1 cKO, n=8). (h‐j) LV tissue sections of WT and *Glud1* ^i^ ^ΔMac^ mice stained with Evans Blue/TTC to assess myocardial infarction area post‐IR. AAR and the IF/AAR ratio were quantified (n=7 per group). Graphs show mean ± SEM. All experiments were independently replicated at least in triplicate. Statistical analyses were conducted using the Mann‐Whitney U test for b and j, using two‐tailed unpaired t‐test for c,i and two‐way ANOVA followed by Tukey's test for d,g. ^*^ *P* < 0.05, ^**^ *P* < 0.005, ***P < 0.0005, N.S., not significant (*P* > 0.05).

To investigate whether *Glud1* deficiency in resident or recruited macrophages contributes to aggravated cardiac injury after IR, we performed bone marrow transplantation (BMT) using bone marrow from WT or *Glud1* ^ΔMo^ donor mice into CD68 eGFP reporter recipient mice, as previously described [26]. Busulfan conditioning was used to reconstitute the bone marrow compartment with donor‐derived cells while preserving tissue‐resident macrophages [28]. In these chimeric mice, donor‐derived recruited macrophages were identified as eGFP negative (eGFP^−^) cells, whereas recipient‐derived resident macrophages retained eGFP expression. Six weeks after BMT, GLUD1 expression was assessed in recipient mice to confirm efficient bone marrow reconstitution (Figure S3c). Following IR injury, chimeric mice reconstituted with donor bone marrow exhibited marked infiltration of donor‐derived eGFP^−^CD68^+^ recruited macrophages into cardiac tissue (Figure S3d,e). Notably, mice reconstituted with *Glud1* ^ΔMo^ donor cells displayed a more pronounced decline in cardiac function than control chimeras (Figure S3f,g, Table S2). Because *Glud1* deletion in this model was confined to donor‐derived recruited macrophages, these results indicate that GLUD1 deficiency in the recruited macrophage compartment is sufficient to worsen cardiac outcome after IR injury. Collectively, these findings demonstrate that the absence of GLUD1 in monocytes/macrophages significantly exacerbates myocardial injury and worsens cardiac function following IR injury.

### 2.3 GLUD1‐Deficient Macrophages Orchestrate a Shift Toward a Pro‐Inflammatory Cardiac Immune Environment and Exacerbate Oxidative Stress Post‐IR Injury

To further elucidate the impact of macrophage‐specific GLUD1 deficiency on cardiac immune homeostasis and oxidative stress, we investigated the immune landscape and redox balance following myocardial IR injury. Macrophages play a pivotal role in orchestrating inflammatory responses and reactive oxygen species (ROS) homeostasis during IR injury, both of which critically contribute to early myocardial damage [11, 18]. Given this, we hypothesized that GLUD1 deficiency in macrophages would disrupt the cardiac immune environment and exacerbate oxidative stress.

We first assessed whether GLUD1 deficiency altered the composition of the cardiac macrophage compartment by flow cytometry. Following IR injury, both WT and *Glud1* ^ΔMo^ mice exhibited an increased proportion of CD45^+^CD11b^+^F4/80^+^ monocytes/macrophages, representing the total monocyte/macrophage population. However, the abundance of total monocytes/macrophages was comparable between the two genotypes, suggesting that GLUD1 deficiency affects macrophage composition rather than the extent of cardiac monocyte/macrophage accumulation (Figure 3a, Figure S3h). In both genotypes, IR injury was associated with a reduced proportion of Tim4^+^Ly6C^−^ tissue‐resident macrophages and an increased proportion of Ly6C^+^ recruited monocytes/macrophages, consistent with the progressive replacement of resident macrophages by recruited cells. Notably, this resident‐to‐recruited shift was more pronounced in *Glud1* ^ΔMo^ mice, which showed a significantly higher proportion of Ly6C^+^ recruited monocytes/macrophages than in WT controls, whereas the Tim4^+^Ly6C^−^ resident macrophage fraction remained comparable between genotypes. No significant differences were observed between WT and *Glud1* ^ΔMo^ mice in sham‐operated groups (Figure 3b,c and Figure S3h).

**FIGURE 3:**
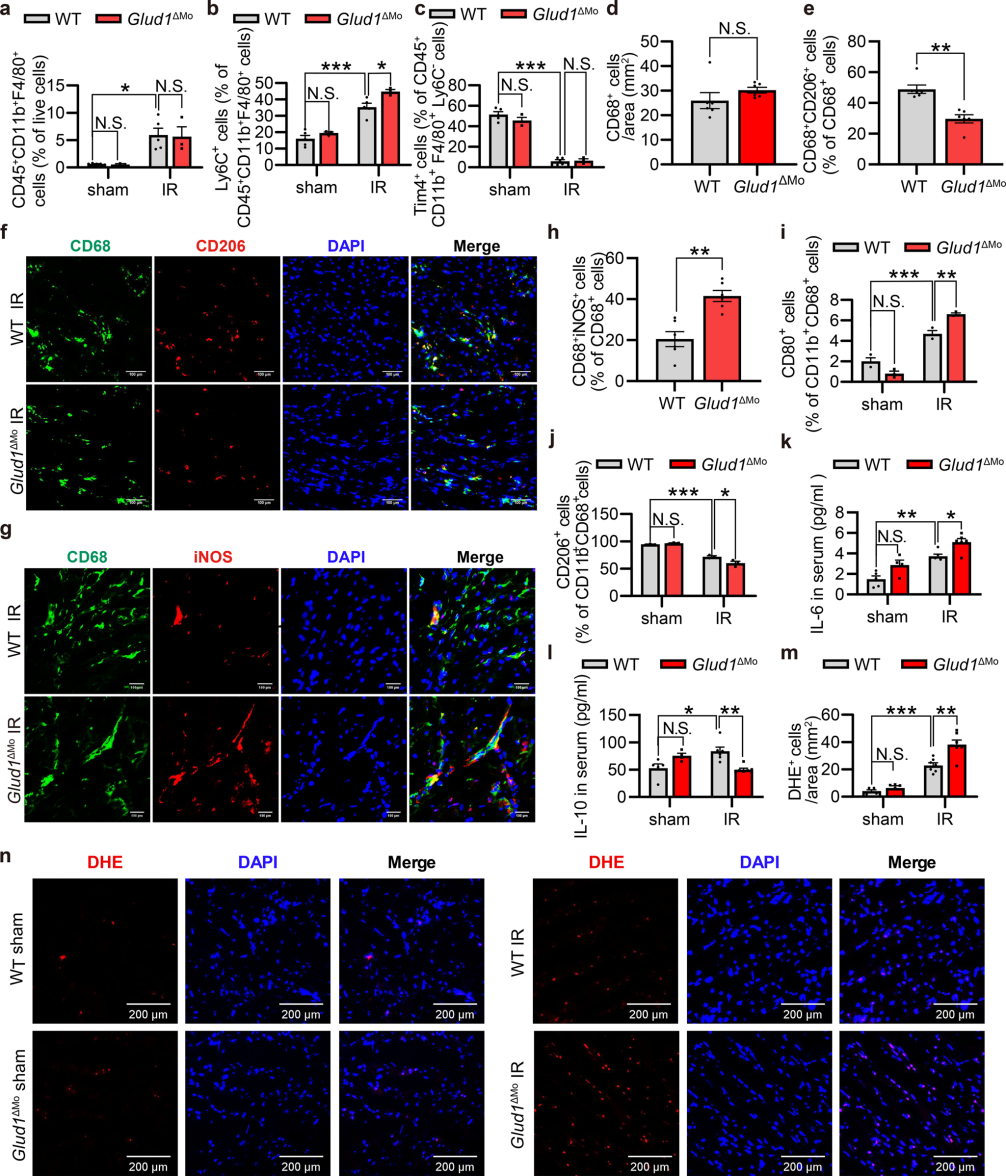
GLUD1‐deficient macrophages orchestrate a shift toward a pro‐inflammatory cardiac immune environment and exacerbate oxidative stress post‐IR injury. (a‐c) Flow cytometric analysis of cardiac CD45^+^CD11b^+^F4/80^+^ monocyte/macrophages, Ly6C^+^ monocyte/macrophages and tissue‐resident Tim4^+^Ly6C^−^ macrophages within the CD45^+^CD11b^+^F4/80^+^ monocyte/macrophage population from WT and *Glud1* ^ΔMo^ mice 24 h post‐IR injury (WT, n=5 per group; *Glud1* ^ΔMo^, n=3 per group). (d‐f) Quantification and representative images of CD68^+^ cells and the percentage of CD206^+^ CD68^+^ cells in CD68^+^ cells (n=6 per group). Scale bar, 100 µm. (g‐h) Representative images and quantification of the percentage of iNOS^+^CD68^+^ cells in CD68^+^ cells (n=6 per group). Scale bar, 100 µm. (i‐j) Flow cytometric analysis of pro‐inflammatory CD80^+^ and anti‐inflammatory CD206^+^ monocyte/macrophages within CD45^+^CD11b^+^CD68^+^ monocyte/macrophages from WT and *Glud1* ^ΔMo^ mice 24 h post‐IR injury (WT sham, n=3; WT IR, n=4; *Glud1* ^ΔMo^, n=3 per group). (k‐l) Serum levels of IL‐6 and IL‐10 in mice 24 h after sham (IL‐6, WT, n=5; IL‐6, *Glud1* ^ΔMo^, n= 4; IL‐10, WT, n=5; IL‐10, *Glud1* ^ΔMo^, n=4) or IR surgery (IL‐6, WT, *Glud1* ^ΔMo^, n=6; IL‐10, WT, n=6; IL‐10, *Glud1* ^ΔMo^, n=7) detected by ELISA (n=4‐7 for each group). (m‐n), Quantification and representative images of dihydroethidium (DHE) positive cells in heart tissues of WT and *Glud1* ^ΔMo^ mice, both sham‐operated (n=5 per group) and IR (n=7 per group). Scale bar, 200 µm. Graphs show mean ± SEM. All experiments were independently replicated at least in triplicate. Statistical analyses were conducted using Mann‐Whitney U test for d,e, two tailed unpaired t‐test for h, and two‐way ANOVA followed by Tukey's test for a‐c and i m. ^*^ *P* < 0.05, ^**^ *P* < 0.005, ^***^ *P* < 0.0005, N.S., not significant (*P* > 0.05).

We next characterized the macrophage landscape in the injured heart by immunofluorescence and flow cytometry. Cardiac sections were stained for CD206 to identify anti‐inflammatory monocytes/macrophages, which are crucial for wound repair and tissue remodeling [18]. Consistent with the flow cytometry data, the total number of CD68^+^ cardiac monocytes/macrophages was comparable in WT and *Glud1* ^ΔMo^ mice (Figure 3d). However, after IR injury, *Glud1* ^ΔMo^ mice exhibited a significant reduction in CD206^+^ monocytes/macrophages compared to WT mice, indicating a diminished anti‐inflammatory response (Figure 3e,f). In contrast, *Glud1* ^ΔMo^ mice showed a marked increase in iNOS^+^ pro‐inflammatory monocytes/macrophages compared to WT controls post‐IR injury (Figure 3 g,h). These observations suggest that GLUD1 deficiency skews macrophage polarization toward a pro‐inflammatory phenotype, impairing the tissue repair processes. Flow cytometry analysis further supported these findings, revealing significant changes in macrophage populations post‐IR injury. While both WT and *Glud1* ^ΔMo^ mice exhibited increased pro‐inflammatory monocyte/macrophage populations following IR injury, *Glud1* ^ΔMo^ mice demonstrated a significant rise in CD80^+^ pro‐inflammatory monocytes/macrophages and a dramatic decrease in CD206^+^ anti‐inflammatory monocytes/macrophages compared to WT controls (Figure 3i,j, Figure S4a,b). Importantly, these differences were not observed in sham‐operated mice, where both CD80^+^ and CD206^+^ monocyte/macrophage populations were comparable in both genotypes (Figure 3i,j). This shift in macrophage phenotype reshapes the cardiac immune environment, as anti‐inflammatory macrophages secrete cytokines that regulate both innate and adaptive immune responses [29]. To further assess changes in myocardial immune homeostasis, we collected postoperative blood from the carotid arteries of mice and conducted ELISA assays to measure serum levels of cytokines. Consistent with the observed macrophage polarization shift, the pro‐inflammatory cytokine IL‐6 levels were elevated in the serum following IR injury, with a more pronounced increase observed in *Glud1* ^ΔMo^ mice compared to WT controls. Conversely, IR injury triggered an increase in the anti‐inflammatory cytokine IL‐10 levels in WT mice, but this elevation was attenuated in *Glud1* ^ΔMo^ mice (Figure 3k,l). In addition to immune dysregulation, we assessed oxidative stress, a critical contributor to myocardial damage during IR injury. Reperfusion following ischemia is well known to trigger ROS accumulation, which exacerbates tissue injury and inflammation [2]. Remarkably, *Glud1* ^ΔMo^ mice exhibited significantly higher ROS levels post‐IR injury compared to WT controls (Figure 3m,n). Collectively, these findings indicate that GLUD1 deficiency in macrophages skews the cardiac immune environment toward a pro‐inflammatory state while simultaneously amplifying oxidative stress.

### 2.4 GLUD1 Deficiency in Macrophages Exacerbates Cardiomyocyte Apoptosis Upon IR Injury In Vitro

To investigate how macrophage GLUD1 deficiency responds to oxidative stress during IR injury and its impact on cardiomyocyte behaviour and biology, potentially aggravating IR injury, we conducted a series of experiments. To assess how BMDMs respond to ROS under IR‐like conditions, BMDMs were isolated from *Glud1* ^ΔMo^ and WT mice, then treated with 150 µM H_2_O_2_ for 8 h or subjected to hypoxia/reoxygenation (HR) [30, 31]. HR was simulated by culturing BMDMs under 1% oxygen for 12 h, followed by reoxygenation for another 12 h. Flow cytometry analysis using the MitoSOX dye revealed a significant increase in ROS accumulation in WT macrophages following HR treatment compared to normoxic conditions (Figure S4c). Conditioned media (CM) were prepared from BMDMs, and neonatal rat ventricular myocytes (NRVMs) were pre‐treated with the CM for 24 h. Subsequently, NRVMs were subjected to in vitro simulated ischemia/reperfusion (sI/R), consisting of 3 h of hypoxia followed by 3 h of reoxygenation to mimic in vivo IR injury conditions (Figure 4a). As expected, sI/R significantly increased apoptosis in NRVMs compared to normoxic conditions. Notably, NRVMs cultured with CM from GLUD1 KO macrophages exhibited comparable levels of apoptosis to those cultured with WT CM under normoxia, indicating that GLUD1 deficiency did not affect baseline apoptosis. However, CM from both HR‐ and H_2_O_2_‐treated GLUD1 KO macrophages markedly exacerbated apoptosis in NRVMs following sI/R compared to WT CM under the same conditions (Figure 4b‐e, Figure S4d‐f). These findings suggest that GLUD1 deficiency in macrophages amplifies oxidative stress, creating a microenvironment that worsens cardiomyocyte apoptosis during IR injury. To eliminate potential effects of external H_2_O_2_ on NRVMs, subsequent experiments focused on the HR‐stimulated condition to induce ROS in BMDMs. ROS accumulation in NRVMs was assessed using the CM‐H2DCFDA probe and dihydroethidium (DHE) staining. The results revealed that sI/R induced ROS accumulation in NRVMs was further exacerbated by CM from GLUD1 KO macrophages compared to WT CM following sI/R (Figure 4f‐h). Oxidative damage in NRVMs was further evaluated by detecting 8‐hydroxy‐2'‐deoxyguanosine (8‐OHdG), showing that CM from GLUD1 KO macrophages significantly increased sI/R‐induced oxidative damage in NRVMs compared to WT CM (Figure 4i). This increase in oxidative damage further underscores the detrimental impact of GLUD1 deficiency in macrophages on cardiomyocytes under IR injury conditions. Furthermore, the protein levels of cleaved caspase‐3 were assessed. Cleaved caspase‐3 levels were comparable among control NRVMs and those cultured with either WT CM or KO CM under normoxia, indicating no significant difference in basal apoptosis. However, cleaved caspase‐3 levels were significantly elevated post sI/R, with a further increase observed in NRVMs cultured with GLUD1 KO CM compared to those with WT CM under sI/R stimulation (Figure 4j‐k). Collectively, these results indicate that enhanced ROS accumulation in GLUD1 KO macrophages creates a less favorable microenvironment, impairing cardiomyocyte recovery and exacerbating IR injury.

**FIGURE 4:**
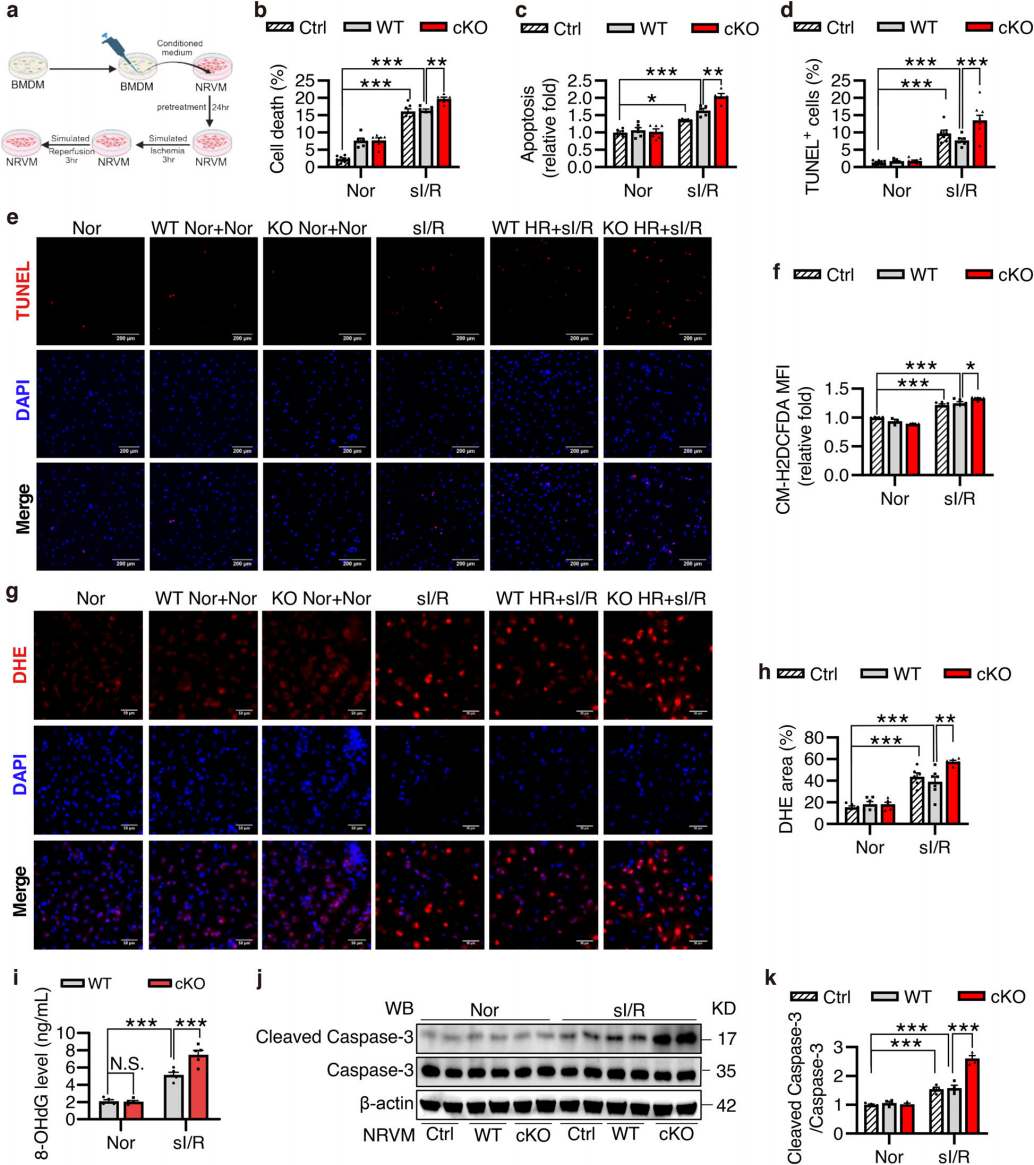
GLUD1 deficiency in macrophages exacerbates cardiomyocyte apoptosis upon I/R in vitro. (a) Schematic representation of the simulated ischemia/reperfusion (sI/R) treatment applied to neonatal rat ventricular myocytes (NRVMs) cultured with conditioned medium (CM) from BMDMs subjected to hypoxia and reoxygenation (HR) stimulation. Figure created in BioRender, Jin, T. (2026) q34w608. (b) Lactate dehydrogenase (LDH) levels in the NRVMs under normoxic (Nor) conditions (Ctrl, n=8; WT, n=6; cKO, n=6) or sI/R treatment (Ctrl, WT, n=7; cKO, n=6). (c) Quantification of NRVM apoptosis by flow cytometry using Annexin V and PI double staining under normal conditions (n=6 per group) or post‐sI/R treatment (n=5 per group). (d‐e) Quantification and representative images of TUNEL staining on NRVMs under normal conditions (Ctrl, n=8; WT, n=7; cKO, n=8) or post‐sI/R treatment (Ctrl, n=7; WT, n=6; cKO, n=9). Scale bar, 200 µm. (f) Cellular ROS levels in NRVMs detected by flow cytometry using CM‐H2DCFDA probe under normal conditions (Ctrl, Nor, n=4; WT, Nor, n=3; cKO, Nor, n=3) or following sI/R treatment (n=6 per group). (g‐h) Representative images and quantification of DHE^+^ NRVMs under normal conditions (n=6 per group) or post‐sI/R treatment (Ctrl, n=6; WT, n=7; cKO, n=6). Scale bar, 50 µm. (i) Levels of 8‐OHdG measured in NRVMs cultured with CM from BMDMs under normal conditions or following sI/R treatment (n=6 per group). (j‐k) Western blot analysis of Caspase‐3 and Cleaved Caspase‐3 expression in NRVMs post‐sI/R stimulation. β‐actin was used as a loading control. Numbers indicate densitometric fold change relative to β‐actin (n=4 per group). Graphs show mean ± SEM. All experiments were independently replicated at least in triplicate. Statistical analyses were conducted using two‐way ANOVA, followed by Tukey's test for b‐d,f,h,i,k. ^*^ *P* < 0.05, ^**^ *P* < 0.005, ^***^ *P* < 0.0005, N.S., not significant (*P* > 0.05).

### 2.5 GLUD1 Deficiency in Macrophages Induces Pro‐Inflammatory Responses and Reprograms Glycolytic Metabolism

To understand how GLUD1 deficiency in macrophages modulates the immune environment during IR injury, we first examined GLUD1 expression after HR exposure in vitro. HR significantly increased GLUD1 expression in WT macrophages (Figure S5a). We next collected conditioned media (CM) from WT and GLUD1 KO macrophages cultured under both normoxic and HR conditions and analyzed them by ELISA. In WT macrophages, HR treatment significantly enhanced the release of pro‐inflammatory cytokines IL‐6 and TNFα. While no notable differences were observed between WT and GLUD1 KO macrophages under normoxia, GLUD1 KO macrophages exhibited a significantly higher release of IL‐6 and TNFα following HR treatment (Figure 5a,b). Consistent with these findings, GLUD1 KO macrophages showed significantly elevated mRNA levels of *Il6*, *Tnf*, *Nos2*, *Cd80* and *Cxcl10* in response to HR treatment (Figure 5c‐g). In contrast, the expression of *Mrc1*, *Arg1*, *Ccl17* and *Il10* was reduced following HR treatment, whereas the baseline expression of these genes remained comparable between WT and GLUD1 KO macrophages (Figure 5h‐i, Figure S5b,c). At the protein level, iNOS expression was elevated, whereas CD206 expression was reduced in GLUD1 KO macrophages compared to WT controls under HR conditions (Figure 5j‐l). The results under normoxic conditions align with our previous study on macrophage GLUD1 during muscle regeneration following cardiotoxin administration [26], where pro‐inflammatory and anti‐inflammatory clusters were comparable in WT and GLUD1 KO macrophages at baseline (Figure 5a‐l, Figure S5b,c). However, upon HR stimulation, macrophages adopted a pronounced pro‐inflammatory phenotype, which was further exacerbated in GLUD1 KO macrophages compared to WT controls (Figure 5a‐l, Figure S5b,c).

**FIGURE 5:**
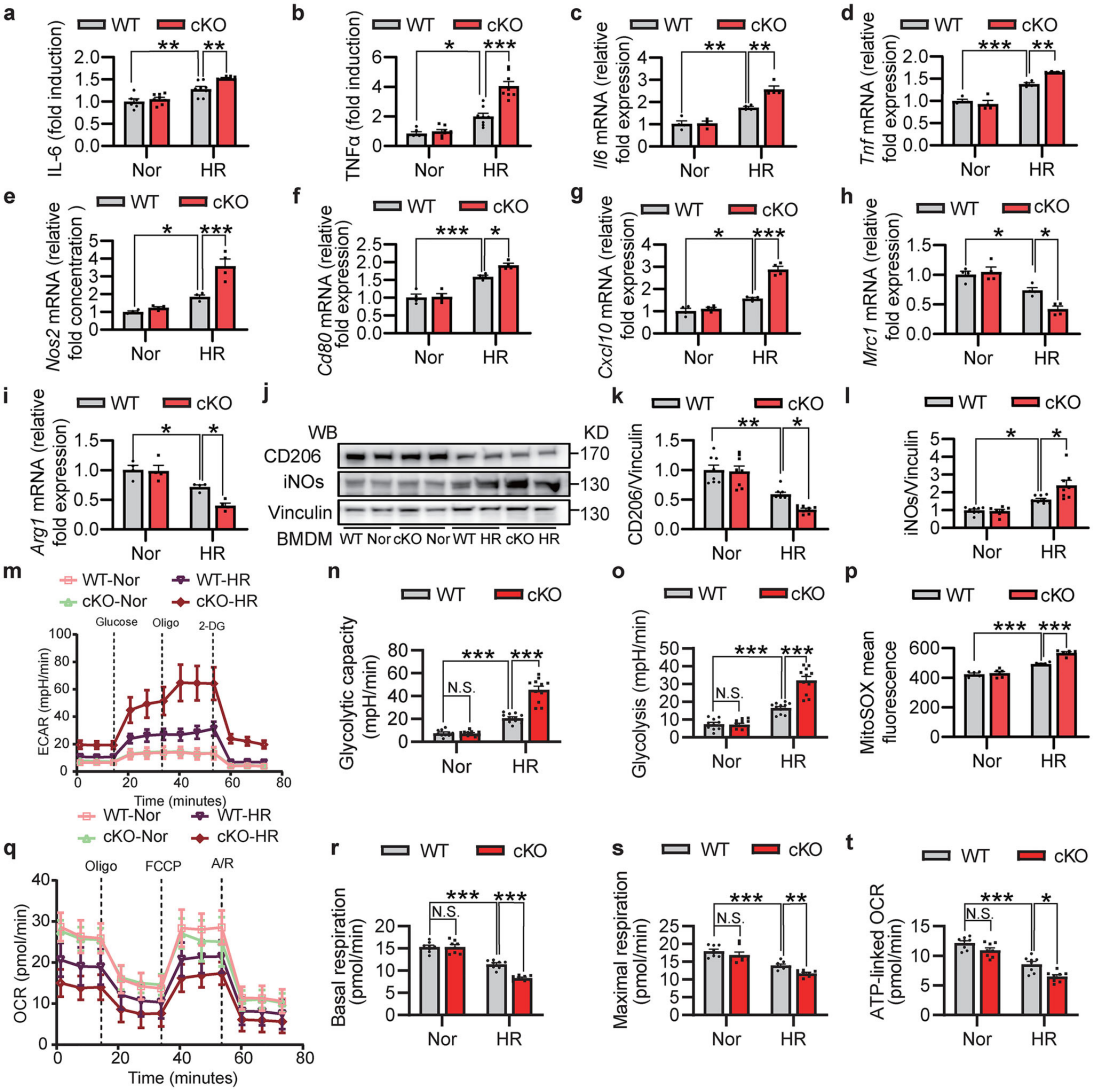
GLUD1 deficiency in macrophages induces pro‐inflammatory responses and reprograms glycolytic metabolism. (a‐b) Levels of pro‐inflammatory cytokines IL‐6 and TNFa in BMDMs medium detected by ELISA under normoxia (WT, n=6; cKO, n=8) and post HR treatment (WT, n=8; cKO, n=8). (c‐i) RT‐qPCR analysis of pro‐inflammatory genes *Il6*, *Tnf*, *Nos2*, *Cd80*, *Cxcl10* and anti‐inflammatory gene *Mrc1*, *Arg1* in BMDMs under normoxia and post‐HR treatment (n=4 per group). (j‐l) Western blot for CD206 and iNOS in BMDMs under normoxia and HR treatment. Vinculin was used as a loading control. Numbers represent densitometric fold change relative to vinculin (n=8 per group). (m‐o) Extracellular acidification rate (ECAR) in BMDMs under normoxia and post‐HR treatment. Glycolytic capacity and glycolysis were analyzed in WT and GLUD1 KO BMDMs (WT, Nor, n=10; WT, HR, n=12; KO, n=11 per group). (p) Mitochondrial ROS levels in WT and GLUD1 KO BMDMs were assessed by flow cytometry using MitoSOX (n=6 per group). (q‐t) Mitochondrial respiration measurement of WT and GLUD1 KO BMDMs under normoxia and post‐HR treatment (n=8 per group). Basal respiration, maximal respiration and ATP‐linked OCR levels were analyzed. Graphs show mean ± SEM. All experiments were independently replicated at least in triplicate. Statistical analyses were conducted using two‐way ANOVA followed by Tukey's test for a‐i,k,l,n‐p,r‐t. ^*^ *P* < 0.05, ^**^ *P* < 0.005, ^***^ *P* < 0.0005, N.S., not significant (*P* > 0.05).

Pro‐inflammatory macrophages reprogram their metabolism toward glycolysis at the expense of oxidative phosphorylation (OXPHOS) [32, 33]. Given that GLUD1 KO macrophages adopt a pro‐inflammatory phenotype, we next investigated how GLUD1 contributes to macrophage metabolism reprogramming. Consistent with our previous findings under normoxic conditions [26], the extracellular acidification rate (ECAR), an indicator of glycolysis, was comparable between the two groups under normoxia. However, HR stimulation significantly increased glycolysis in macrophages, and this effect was further amplified in GLUD1 KO macrophages compared to WT controls (Figure 5m‐o). Flow cytometry analysis using MitoSOX dye indicated a significant increase in mitochondrial ROS (mitoROS) accumulation in GLUD1 KO macrophages following HR treatment (Figure 5p), consistent with previous studies showing that the glycolytic switch in inflammatory macrophages is accompanied by succinate dehydrogenase (SDH)‐driven reactive oxygen species (ROS) production [34]. Additionally, HR treatment reduced the oxygen consumption rate (OCR) in macrophages. GLUD1 deficiency further suppressed OCR in macrophages under HR conditions despite no differences between the two groups under normoxia (Figure 5q‐t). Integrating these findings with our previous in vivo results, we conclude that the absence of GLUD1 in macrophages reprograms their metabolism toward glycolysis, driving an increased release of pro‐inflammatory cytokines and augmented mitoROS accumulation in myocardial IR injury.

### 2.6 GLUD1 Deficiency in Macrophages Activates NF‐κB via the mitoROS/PHD2 Axis Following HR

Next, we aimed to investigate how the loss of GLUD1 in macrophages promotes pro‐inflammatory responses in cardiac IR injury. The results of RNA‐seq analysis of monocytes from AMI patients post interventional therapy, compared to healthy controls, underscored the importance of HIF‐1α and NF‐κB signaling in IR injury (Figure S1h). MitoROS inactivates prolyl hydroxylase domain proteins (PHDs) [35] through iron oxidation, thus stabilizing hypoxia‐inducible factors (HIFs) and activating nuclear factor‐κB (NF‐κB) [11, 34, 36, 37]. First, we observed that GLUD1 KO macrophages exhibited increased levels of HIF‐1α following HR stimulation compared to WT macrophages (Figure 6a,b). Additionally, GLUD1 deficiency was associated with reduced protein levels of the NF‐κB inhibitor IκBα, resulting in increased phosphorylation of NF‐κB p65 compared to WT macrophages following HR stimulation (Figure 6a,c,d). Phosphorylation of IκBα by IκB kinase (IKK) promotes its ubiquitination and degradation and the direct phosphorylation of p65 at Ser536 leads to its nuclear translocation and transcriptional activity of NF‐κB [38, 39]. Consistently, HR treatment induced the nuclear translocation of NF‐κB p65 in both WT and, to a greater extent, GLUD1 KO macrophages, as detected by WB and immunofluorescence staining (Figure 6e‐h), indicating enhanced NF‐κB activation.

**FIGURE 6:**
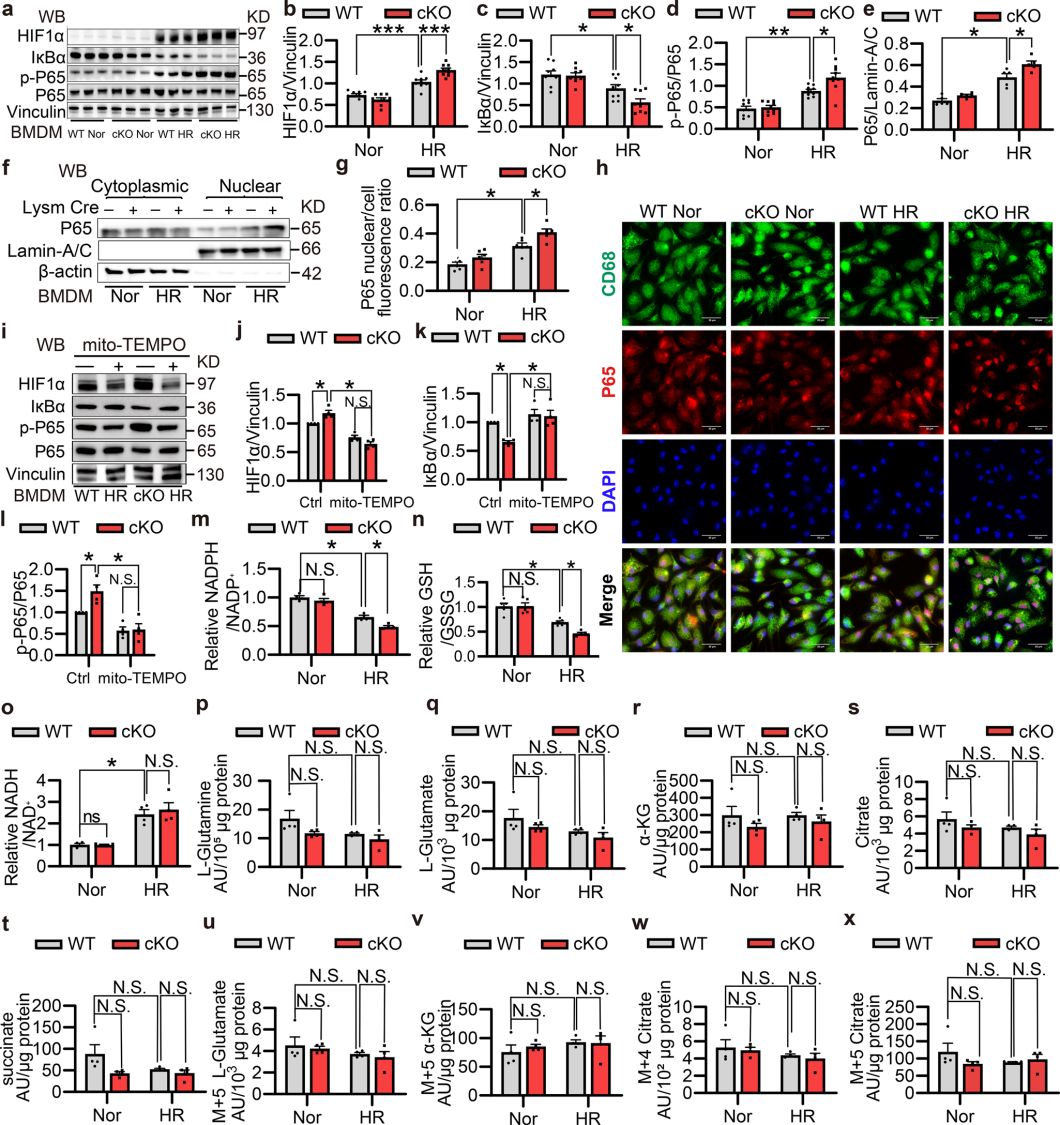
GLUD1 deficiency in macrophages activates NF‐kB via the mitoROS/PHD2 axis following HR. (a‐d) Western blot and quantification of HIF1a, IĸBa, p‐P65, P65 in WT and GLUD1 KO BMDMs under normoxia and HR treatment (n=9 per group). (e‐f) Western blot and quantification of P65 in cytosolic and nuclear fractions of WT and GLUD1 KO BMDMs under normoxia and HR treatment (n=5 per group). β‐actin and Lamin A/C as loading controls, respectively. (g‐h) Representative images and quantification of P65 staining in the nucleus and cytoplasm. The ratio of P65 in the nucleus to the cytoplasm was quantified (n=6 per group). Scale bar, 20 µm. (i‐l) Western blot and quantification of HIF1a, IĸBa, p‐P65, P65 in WT and GLUD1 KO BMDMs treated with MitoTEMPO (mitochondrial ROS scavenger) (n=4 per group) post HR treatment. Vinculin was used as a loading control. Numbers represent densitometric fold change relative to vinculin. (m‐o) Levels of NADPH/NADP^+^ (n=4 per group) GSH/GSSG (n=5 per group) NADH/NAD^+^ ratio (n=4 per group) in WT and GLUD1 KO BMDMs under normoxia and HR stimulation. (p‐t) Liquid chromatography‐mass spectrometry‐based quantification of glutamine, glutamate, a‐KG, citrate, and succinate in WT and GLUD1 KO BMDMs under normoxia or HR stimulation (n = 4 per group). (u‐x) Fractional enrichment of the M+5 glutamate, M+5 a‐KG, M+4 citrate, and M+5 citrate following [^13^C_5_]‐glutamine tracing in WT and GLUD1 KO BMDMs under normoxia or HR stimulation (n = 4 per group). Graphs show mean ± SEM. All experiments were independently replicated at least in triplicate. Statistical analyses were conducted using two‐way ANOVA followed by Tukey's test for b‐e,g,j‐x. ^*^ *P* < 0.05, ^**^ *P* < 0.005, ^***^ *P* < 0.0005, N.S., not significant (*P* > 0.05).

To investigate the role of mitoROS in HIF‐1α and NF‐κB activation in GLUD1 KO macrophages, we treated macrophages with mito‐TEMPO, a mitochondrially targeted ROS scavenger [40]. Mito‐TEMPO inhibited HR‐induced nuclear translocation of NF‐κB p65 in WT macrophages, indicating reduced NF‐κB activity (Figure S5d,e). Furthermore, mito‐TEMPO effectively suppressed HIF‐1α accumulation, IκBα degradation and subsequent NF‐κB p65 phosphorylation in GLUD1 KO macrophages following HR stimulation (Figure 6i‐l). These results underscore the critical role of mitoROS in triggering HIF‐1α accumulation and NF‐κB activation in GLUD1 KO macrophages. Among the three PHD family members, PHD2 is the key isoenzyme in macrophages that negatively regulates HIF‐1α accumulation and NF‐κB activity through protein regulation of IKKβ/IκBα [41]. Thus, we assessed deferoxamine (DFO), an iron chelator that inhibits prolyl hydroxylase activity by sequestering its iron cofactor [42], as a possible way to mimic in a WT context what was seen in GLUD1 KO macrophages during HR. In line with our previous observations, HR treatment resulted in enhanced HIF‐1α accumulation and IκBα degradation, which leads to enhanced phosphorylation of NF‐κB p65 in GLUD1 KO macrophages compared to WT controls. DFO treatment further promoted HIF‐1α levels and IκBα degradation, thus enhancing nuclear translocation and the phosphorylation of p65 in WT macrophages to levels comparable to those in GLUD1 KO macrophages following HR stimulation (Figure S5d‐i), indicating the contribution of PHD activity in HIF‐1α accumulation and NF‐κB activation in GLUD1 KO macrophages. To further dissect the roles of HIF‐1α and NF‐κB in this process, we used chetomin, a specific inhibitor of HIF‐1α that disrupts the interaction between the transcriptional coactivator p300 and the HIF‐1α alpha subunit [35, 43], along with NF‐κB‐IN, a specific inhibitor of NF‐κB that blocks IKK activity [44], thereby preventing IκBα degradation and inhibiting NF‐κB activation. The efficiency of chetomin and NF‐κB‐IN was validated by their respective effects on reducing HIF‐1α accumulation and IκBα degradation (Figure S5f‐i). Chetomin effectively suppressed HIF‐1α accumulation, IκBα degradation and NF‐κB p65 phosphorylation, bringing them to comparable levels in both WT and GLUD1 KO macrophages under HR stimulation (Figure S5f‐i). Consistently, NF‐κB‐IN inhibited IκBα degradation and p65 phosphorylation in GLUD1 KO macrophages to levels comparable to WT controls, without affecting the higher levels of HIF‐1α observed in GLUD1 KO macrophages (Figure S5f‐m). Collectively, these findings demonstrate that elevated mitoROS in GLUD1 KO macrophages inhibits PHD2 activity, leading to HIF‐1α accumulation, and promotes IκBα degradation, and NF‐κB activation.

Since GLUD1 utilizes NADP^+^ or NAD^+^ as cofactors, we measured the NADPH/NADP^+^, NADH/NAD^+^, and GSH/GSSG ratios in WT and GLUD1 KO macrophages. Following HR stimulation, GLUD1 KO macrophages showed significantly lower NADPH/NADP^+^ and GSH/GSSG ratios compared to WT macrophages, while the NADH/NAD^+^ ratio remained comparable between genotypes (Figure 6m‐o). This decrease in NADPH/NADP^+^ and GSH/GSSG ratios in GLUD1 KO macrophages was accompanied by reduced ferrous iron (Fe^2+^) levels after HR (Figure S6a). Because PHD enzymes require catalytic iron in the reduced ferrous state, the impaired ability to maintain the Fe^2+^ pool suggests an iron‐dependent mechanism by which the GLUD1‐associated redox imbalance suppresses PHD2 activity [45]. This observation is consistent with our DFO experiments, in which limitation of available iron exaggerated the HIF‐1α/NF‐κB response in WT macrophages.

Because α‐ketoglutarate (α‐KG) and succinate can regulate PHD activity, we next investigated whether the redox and inflammatory phenotypes resulted from altered glutamine‐derived carbon metabolism. Levels of glutamine, glutamate, α‐KG, citrate, and succinate were comparable between WT and GLUD1 KO macrophages at baseline and after HR (Figure 6p‐t). Consistently, [^13^C_5_]‐glutamine tracing showed unchanged enrichment of glutamate (M+5) and α‐KG (M+5) in WT and GLUD1 KO macrophages under both conditions (Figure 6u,v). Citrate (M+4) labeling, reflecting oxidative glutamine carbon flux through the TCA cycle, was also unchanged. In addition, citrate (M+5) labeling was not increased, providing no evidence for enhanced reductive carboxylation (Figure 6w,x). These data indicate that glutamine‐derived carbon contribution to α‐KG is preserved despite GLUD1 loss, likely through compensatory aminotransferase activity, as previously reported [46]. Indeed, the cytosolic branched‐chain amino acid aminotransferase 1 (*Bcat1*) was significantly upregulated in GLUD1 KO macrophages, whereas the mitochondrial isoform *Bcat2* and the glutamate oxaloacetate transaminases *Got1* and *Got2* remained unchanged (Figure S6b). BCAT1 catalyses the reversible transamination between glutamate and α‐KG, with the net direction of this reaction determined by the relative availability of its substrates [47, 48]. Silencing *Bcat1* reduced α‐KG and succinate levels after HR (Figure S6c‐e), supporting a role for BCAT1 in maintaining α‐KG and downstream TCA metabolites in the absence of GLUD1.

To test whether this residual carbon supply drives HIF‐1α/NF‐κB activation, GLUD1 KO macrophages were supplemented with cell‐permeable dimethyl α‐ketoglutarate (DM‐αKG) or dimethyl succinate (DM‐succ) at viability‐preserving concentrations (Figure S6f‐i). DM‐αKG supplementation did not attenuate HR‐induced HIF‐1α/NF‐κB activation, and DM‐succ did not further enhance this pathway in GLUD1 KO macrophages (Figure S6j,k). Neither metabolite altered NRVM injury under sI/R conditions (Figure S6l,m), indicating that the exaggerated inflammatory response is not driven by α‐KG or succinate abundance. Although *Bcat1* knockdown reduced α‐KG and succinate levels in GLUD1 KO macrophages, it did not alter the NADPH/NADP^+^ or GSH/GSSG ratios in either genotype under normoxia or HR, and the HR‐induced decline in both ratios persisted after *Bcat1* silencing in GLUD1 KO macrophages (Figure S6n,o). Collectively, these findings indicate that BCAT1 contributes to maintaining glutaminolysis‐derived carbon metabolites in GLUD1 KO macrophages but cannot restore NADP^+^‐dependent NADPH regeneration or GSH redox homeostasis. Thus, GLUD1 deficiency amplifies macrophage pro‐inflammatory responses after HR by activating NF‐κB through the mitoROS/PHD2 axis, independently of α‐KG or succinate abundance.

### 2.7 Pharmacologic Activation of GLUD1 Alleviates Myocardial IR Injury

To explore the therapeutic potential of pharmacologic GLUD1 activation in mitigating myocardial IR injury, we evaluated the effects of the GLUD1 activator BCH [49, 50] in both in vitro and in vivo models. Initially, BMDMs were treated with a range of BCH concentrations to determine the optimal dose for GLUD1 activation. GLUD1 activity was assessed after 24 h of incubation, as previously described [26]. The results revealed that GLUD1 activity peaked at 10 mM BCH (Figure S7a), which was subsequently used for further experiments. CM were collected from BCH‐treated BMDMs following HR stimulation. Notably, BCH‐treated BMDMs significantly reduced mRNA levels of pro‐inflammatory markers, including *Il1b, Il6*, and *Nos2*, compared to vehicle‐treated controls (Figure 7a‐c). BCH treatment also reduced mitoROS accumulation in BMDMs, as measured by MitoSOX fluorescence, highlighting its ability to mitigate oxidative stress in macrophages (Figure 7d). Furthermore, CM from BCH‐treated BMDMs significantly attenuated cell death, apoptosis and ROS accumulation in NRVMs subjected to sI/R stimulation (Figure 7e‐h). These results demonstrate that pharmacologic activation of GLUD1 in macrophages creates a protective microenvironment that improves cardiomyocyte survival and reduces oxidative damage under IR conditions.

**FIGURE 7:**
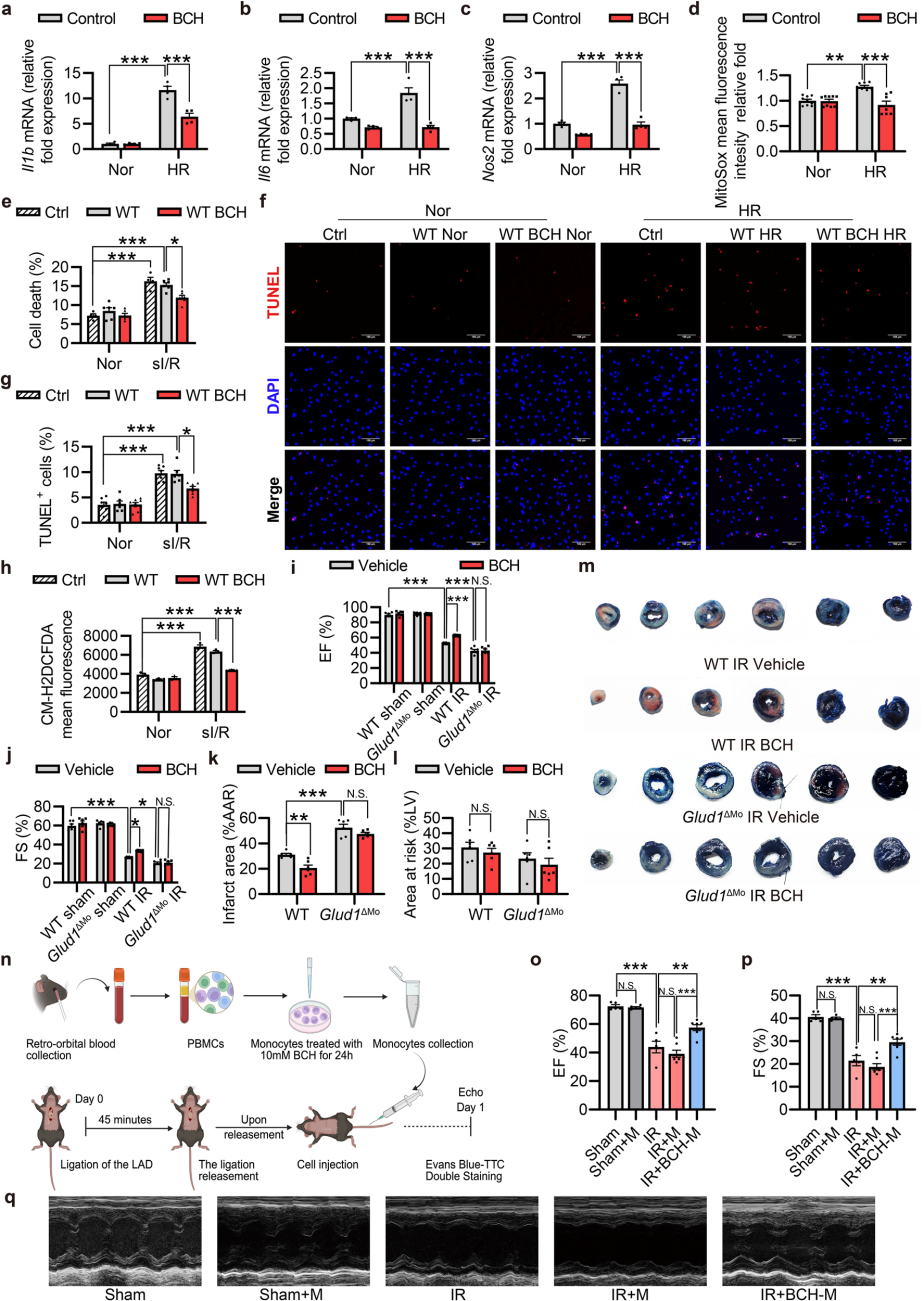
Pharmacologic activation of GLUD1 alleviates myocardial IR injury. (a‐c) RT‐qPCR analysis of pro‐inflammatory genes *Il1b*, *Il6*, and *Nos2* in BMDMs post‐BCH administration (n=4 per group). (d) Mitochondrial ROS was detected in BCH‐treated BMDMs by flow cytometry using MitoSOX (Nor, n=9 per group; HR, Control, n=10; HR, BCH, n=9). (e) Lactate dehydrogenase (LDH) levels in the NRVMs cultured with CM from BCH‐treated BMDMs post‐sI/R treatment (Ctrl, n=4 per group; WT/WT BCH, n=6 per group). (f‐g) Representative images and quantification of TUNEL staining in NRVMs cultured with CM from BCH‐treated BMDMs post‐sI/R treatment (Ctrl, n=7 per group; WT, n=6 per group; WT BCH, Nor, n=8; WT BCH, HR, n=7). Scale bar, 100 µm. (h) Cellular ROS levels were detected in NRVMs cultured with CM from BCH‐treated BMDMs post‐sI/R treatment by flow cytometry using CM‐H2DCFDA (n=3 per group). (i‐j) Quantification of EF and FS in WT and *Glud1* ^ΔMo^ mice treated with BCH or PBS post sham or IR injury (n=6 per group). (k‐m) Representative images and quantification of Evans Blue/TTC‐stained left ventricular sections from both BCH‐ or PBS‐treated WT and *Glud1* ^ΔMo^ mice after IR injury. AAR and the IF/AAR ratio were quantified (n=6 per group). (n) Schematic representation of BCH‐treated monocyte transfusion in IR surgery. Figure created in BioRender, Shang, M. (2026) r7fou9s. (o‐q) Quantitative analysis and representative echocardiography images of EF and FS in mice receiving monocyte transfusions treated with BCH, following sham or IR injury (sham, n=5; sham+M, n=5; IR, n=5; IR+M, n=7; IR+BCH‐M, n=7). Graphs show mean ± SEM. All experiments were independently replicated at least in triplicate. Statistical analyses were conducted using two‐way ANOVA followed by Tukey's test in addition to o,p, where one‐way ANOVA followed by Tukey's test was applied. ^*^ *P* < 0.05, ^**^ *P* < 0.005, ^***^ *P* < 0.0005, N.S., not significant (*P* > 0.05).

Next, we investigated the therapeutic potential of GLUD1 activation in vivo using a mouse model of myocardial IR injury. Sixteen hours after intraperitoneal injection (i.p.) of BCH, peritoneal macrophages (PM) were harvested, and increased GLUD1 activity in these macrophages was confirmed (Figure S7b). Importantly, hematoxylin and eosin staining of liver, kidney, and spleen from the BCH‐treated mice revealed no morphological changes, confirming the safety of BCH administration (Figure S7c). To assess cardiac function, echocardiographic analysis was performed following IR injury. BCH treatment significantly mitigated IR‐induced cardiac dysfunction, as shown by improved ejection fraction and fractional shortening (Figure 7i,j, Figure S7d, Table S3). To evaluate myocardial damage, Evan's Blue‐TTC double staining was used to evaluate the infarct area and area at risk. While the areas at risk were comparable between the groups, BCH treatment resulted in a notable reduction in infarct area during IR injury (Figure 7k‐m). In line with these observations, BCH treatment also significantly reduced IR‐induced cardiac ROS accumulation (Figure S7e,f). Importantly, in *Glud1* ^ΔMo^ mice, which lack macrophage GLUD1, BCH failed to improve cardiac dysfunction, reduce myocardial injury, or suppress IR‐induced cardiac ROS accumulation compared with vehicle treatment, indicating that the protective effect of BCH depends on macrophage GLUD1 (Figure 7i‐m, Figure S7e,f). These results further support the role of GLUD1 activation in mitigating oxidative stress and improving cardiac function during IR injury.

To further demonstrate the protective role of GLUD1 activation in monocyte‐derived macrophages during IR injury, we performed adoptive transfer of peripheral blood‐derived monocytes, as previously described [51, 52, 53]. The purity of the isolated monocytes was verified by flow cytometry, with approximately 84% of cells being CD115^+^ (Figure S7g,h). Monocytes were treated with BCH or vehicle for 24 h to activate GLUD1 and were subsequently injected via the tail vein following IR surgery (Figure 7n). Transfer of vehicle‐treated monocytes did not significantly affect cardiac function or infarct size compared with non‐transferred mice. In contrast, transfer of BCH‐treated monocytes significantly improved cardiac function and reduced the infarct area post‐IR injury (Figure 7o‐q, Table S4, Figure S7i‐k). These findings highlight the critical role of GLUD1 activation in monocyte‐derived macrophages in mitigating IR‐induced myocardial damage and improving cardiac outcomes. Collectively, these data demonstrate that pharmacologic activation of macrophage GLUD1 by BCH creates a protective microenvironment that alleviates myocardial damage and improves cardiac function during IR injury. These findings underscore the therapeutic potential of GLUD1 activation as a novel strategy for reducing IR damage and improving cardiac function during IR injury.

## 3 Discussion

Myocardial ischemia‐reperfusion (IR) injury occurs when blood supply to the infarcted heart is re‐established, triggering a surge in reactive oxygen species (ROS) production [11, 12, 54]. While reperfusion is essential to salvage ischemic tissue, the accompanying oxidative stress and inflammatory responses significantly exacerbate myocardial damage, limiting the therapeutic efficacy of percutaneous coronary intervention (PCI) in patients with acute myocardial infarction. The mechanisms underlying IR injury are complex and multifaceted, with excessive free radical production and overwhelming inflammatory responses serving as pivotal contributors. Increasing evidence highlights the significance of immune cells, particularly macrophages, in restoring cardiac homeostasis following injury [29]. Among the immune cells recruited to the heart, macrophages are predominant and exhibit considerable heterogeneity and functional versatility. By mediating cardiac inflammation, efferocytosis, tissue remodeling, and the restoration of cardiac homeostasis, macrophages play indispensable roles in IR injury [55, 56]. However, the mechanisms by which macrophages regulate reactive oxygen species (ROS) accumulation and orchestrate the immune responses to restore cardiac immune homeostasis during IR injury remains incompletely understood.

Our previous study demonstrated that, independently of immune responses, GLUD1‐deficient macrophages enhance glutamine production and secretion, thereby boosting muscle stem cell activation and muscle regeneration in the context of muscle ischemia, injury or aging [26]. Expanding on this concept, the present study provides novel insights into the role of GLUD1 in cardiac macrophages during myocardial IR injury. Specifically, we observed a significant upregulation of GLUD1 expression in cardiac macrophages during myocardial IR injury in a mouse model, as well as in circulating monocytes from patients with acute myocardial infarction following interventional therapy. Notably, GLUD1 deficiency in macrophages markedly exacerbated IR injury, leading to increased levels of ROS and enhanced cardiomyocyte apoptosis. These observations underscore the essential role of macrophage GLUD1 in maintaining cardiac immune and redox homeostasis during myocardial IR injury.

The heterogeneity of cardiac macrophages is well‐recognized. Cardiac macrophage populations comprise embryonically derived, self‐renewing resident macrophages that maintain tissue homeostasis, as well as monocyte‐derived macrophages recruited upon injury [57, 58, 59]. Following IR injury, cardiac monocytes/macrophages include Lyve1^+^ resident macrophages, interferon‐responsive Isg^+^, MHC‐II^+^ and Mt^hi^ subsets, and monocyte‐derived Blt1^+^ and S100a9^+^ intermediate populations. In this study, Ly6C^+^ monocytes, which exhibited the highest *Glud1* expression, occupied the inferred origin of the monocyte‐to‐macrophage trajectory and preceded the peak of inflammatory activation. This positioning suggests *Glud1* enrichment is an early feature of the recruited macrophage inflammatory response to IR injury, consistent with the higher GLUD1 protein levels detected in sorted Ly6C^+^ and CCR2^+^ cells. Functionally, GLUD1 deficiency expanded the Ly6C^+^ recruited monocyte/macrophage population without altering the composition of the TIM4^+^Ly6C^−^ resident compartment, thereby shifting the cardiac monocyte/macrophage landscape toward a more inflammatory state.

As the most abundant amino acid in the body, glutamine is indispensable for cell growth, maintenance, and metabolism, classifying it as a conditional essential amino acid [60, 61]. Glutamine plays a vital role in cellular bioenergetics, serving as a precursor for nucleotide synthesis, protein biosynthesis, and redox balance. Notably, a high glutamine‐to‐glutamate ratio has been positively correlated with a reduced risk of cardiovascular disease (CVD) [20, 21, 22, 23]. Glutamine is catabolized into mitochondrial α‐ketoglutarate by glutaminase 1 (GLS1) and glutamate dehydrogenase 1 (GLUD1). The administration of glutamine has shown benefits in improving cardiac function following myocardial infarction [62]. However, the role of glutamine metabolism in CVD is complex and context‐dependent. Excess glutamate produced by GLS1 accelerates arterial calcification [63], while GLS1 inhibition prevents cardiac hypertrophy and fibrosis [64]. Furthermore, macrophage‐specific GLS1 deletion impairs efferocytosis and accelerates atherosclerosis [65], while a glutamine‐ and serine‐free diet reduces pathogenic vascular fibroblasts activation in pulmonary hypertension [61]. Despite these advances in understanding the roles of glutamine and GLS1 in CVD, the role of GLUD1 in CVD remains poorly understood. This study addresses this gap by demonstrating that GLUD1 plays a critical role in regulating macrophage function and redox homeostasis during myocardial IR injury.

Beyond its metabolic functions, glutaminolysis orchestrates macrophage activity and function [65, 66, 67]. GLUD1 catalyzes the reversible oxidative deamination of L‐glutamate to produce α‐ketoglutarate, using NADP^+^ or NAD^+^ as coenzymes and generating NADPH or NADH in the process. The ratios of NAD(P)H/NAD(P)^+^ and GSH/GSSG regulate the redox state of the cellular milieu, forming a redox buffer that maintains homeostasis under steady‐state conditions [68]. Consistent with our previous findings, this study demonstrates that GLUD1 does not influence macrophage immune responses under normoxic conditions. Interestingly, however, the deletion of GLUD1 in macrophages led to enhanced pro‐inflammatory responses during IR injury. Mechanistically, GLUD1 deficiency impairs the ROS scavenging capacity of macrophages by reducing the NADPH/NADP^+^ and GSH/GSSG ratios during IR injury. This reduction in antioxidant capacity results in elevated mitochondrial ROS, which in turn inactivates PHD2, stabilizes HIF‐1α expression, and activates the NF‐κB, leading to the release of pro‐inflammatory factors and exacerbating myocardial damage during IR (Graphical Abstract).

Given that α‐KG serves as an essential co‐substrate for PHDs and that succinate inhibits these enzymes, we examined whether altered α‐KG or succinate abundance accounted for the enhanced HIF‐1α/NF‐κB activation in GLUD1 KO macrophages. Glutamine, α‐KG and succinate levels were comparable between WT and GLUD1 KO macrophages at baseline and after HR, and neither DM‐α‐KG nor DM‐succinate altered HR‐induced HIF‐1α/NF‐κB activation in either WT or GLUD1 KO macrophages. Silencing *Bcat1* lowered α‐KG and succinate levels after HR, indicating that a BCAT1‐dependent transamination route sustains these metabolites in the absence of GLUD1. Because transamination is redox‐neutral, this route restores glutamine‐derived carbon flux without regenerating reducing equivalents. Consistent with this, the NADPH/NADP^+^ and GSH/GSSG ratios remained reduced despite preserved α‐KG and succinate pools. GLUD1 KO macrophages also exhibited reduced ferrous iron (Fe^2+^) levels after HR. Because PHD catalysis requires its iron cofactor to be maintained in the reduced Fe^2+^ state, and because the cytosolic labile iron pool is buffered by glutathione, which helps maintain iron in the reduced state [69], the loss of Fe^2+^ suggests an iron‐dependent mechanism linking the redox imbalance caused by GLUD1 deficiency to impaired PHD function. This interpretation is supported by our DFO experiments, in which limiting available iron exaggerated the HIF‐1α/NF‐κB response in WT macrophages, whereas scavenging mitochondrial ROS with mito‐TEMPO reversed the exaggerated response in GLUD1 KO macrophages. Thus, the primary defect caused by GLUD1 deficiency lies in NADPH‐ and GSH‐dependent redox balance rather than in glutamine‐derived carbon supply, resulting in persistent ROS accumulation and an aggravated pro‐inflammatory response. Although the mitoROS/PHD2/HIF‐1α/NF‐κB axis has been well characterized, these findings position macrophage GLUD1 upstream of this axis as a metabolic checkpoint for inflammatory activation during IR injury.

From a therapeutic perspective, the findings of this study highlight the potential of GLUD1 activation as a novel strategy to mitigate myocardial IR injury. The administration of BCH, a known allosteric GLUD1 activator [49, 50], effectively reduced oxidative stress and inflammation in macrophages, thereby alleviating myocardial damage and improving cardiac function after IR injury. These effects were accompanied by enhanced ROS‐scavenging capacity in macrophages, contributing to the restoration of cardiac redox balance and immune homeostasis. Importantly, BCH conferred no protection in monocyte/macrophage GLUD1‐deficient mice, indicating that its beneficial effects depend on macrophage GLUD1. BCH treatment was well tolerated in vivo, with no adverse effects on liver, kidney, or spleen, further supporting its therapeutic potential. Furthermore, adoptive transfer of BCH‐treated monocytes significantly improved cardiac function and reduced infarct size after IR injury, indicating that the protective effect can be mediated through the monocyte compartment. In addition, BCH has been reported to inhibit LAT1 and LAT2 [70, 71]. The loss of efficacy in GLUD1‐deficient mice suggests that inhibition of LAT1/LAT2 is not sufficient to account for the observed cardioprotection. Nevertheless, more selective GLUD1 activators will be required to establish the therapeutic value of this axis and to exclude a potential parallel contribution of LAT1/LAT2 inhibition.

In summary, this study reveals the critical role of macrophage GLUD1 in regulating ROS accumulation and modulating subsequent immune responses during myocardial IR injury. Biologically, this study elucidates how macrophages regulate ROS homeostasis, thereby restoring cardiac redox equilibrium and immune homeostasis. From a therapeutic perspective, the administration of BCH or the transfusion of BCH‐treated monocytes offers a promising clinical strategy to mitigate IR injury. By activating macrophage GLUD1, we potentially preserve cardiac function and reduce IR injury. Therefore, targeting macrophage GLUD1 emerges as a potential therapeutic approach for the treatment of myocardial IR injury.

## Author Contributions

T.J. performed the majority of the experiments, analyzed and interpreted the data, and drafted the manuscript. C.L. conducted the in vitro experiments and contributed to the manuscript writing. Q.Z. performed flow cytometry, histology and mouse treatments. R.W. performed single‐cell RNA sequencing data analysis and interpretation. Y.T. assisted with flow cytometry, the BMT assay, and in vitro assays. W.C. performed RNA sequencing analysis. N.R.,T.Z., X.R., and C.H. assisted with in vitro assays. J.C. performed the IR surgery. R.L. performed the cardiac echocardiography measurement. L.Y. provided critical suggestions and critically revised the manuscript. M.M. provided the two *Glud1* knockout mouse strains, offered critical suggestions, and critically revised the manuscript. W.Z. recruited volunteers and collected blood samples. G.F. critically revised the manuscript. M.S. supervised the project, designed the experiments, interpreted the data, provided scientific direction, and wrote the manuscript.

## Ethics Statement

All experimental animal procedures were approved by the Animal Care and Use Committee of the School of Medicine, Zhejiang University (Approval number: ZJU20230056). All experimental protocols using patient blood samples were approved by Sir Run Run Shaw Hospital, School of Medicine, Zhejiang University (Approval number: 2023‐0253).

## Conflicts of Interest

The authors declare no conflicts of interest.

## Supporting information




**Supporting File 1**: advs78416‐sup‐0001‐SuppMat.pdf.

**Supporting File 2**: advs78416‐sup‐0002‐SuppMat.pdf.

**Supporting File 3**: advs78416‐sup‐0003‐SuppMat.pdf.

**Supporting File 4**: advs78416‐sup‐0004‐SuppMat.docx.

## Data Availability

The data that support the findings of this study are available from the corresponding author upon reasonable request.
