# Supplementary material for: Macrophage GLUD1 is Essential for Redox Balance and Immune Homeostasis in Cardiac Injury
Source: Adv Sci (Weinh). 2026 Oct 28:e78416. Online ahead of print. doi: 10.1002/advs.78416 (PMC13717128; doi:10.1002/advs.78416)
Supplement: Supplementary file 1 [file ADVS-9999-e78416-s001.pdf]

Supplementary Figure 1

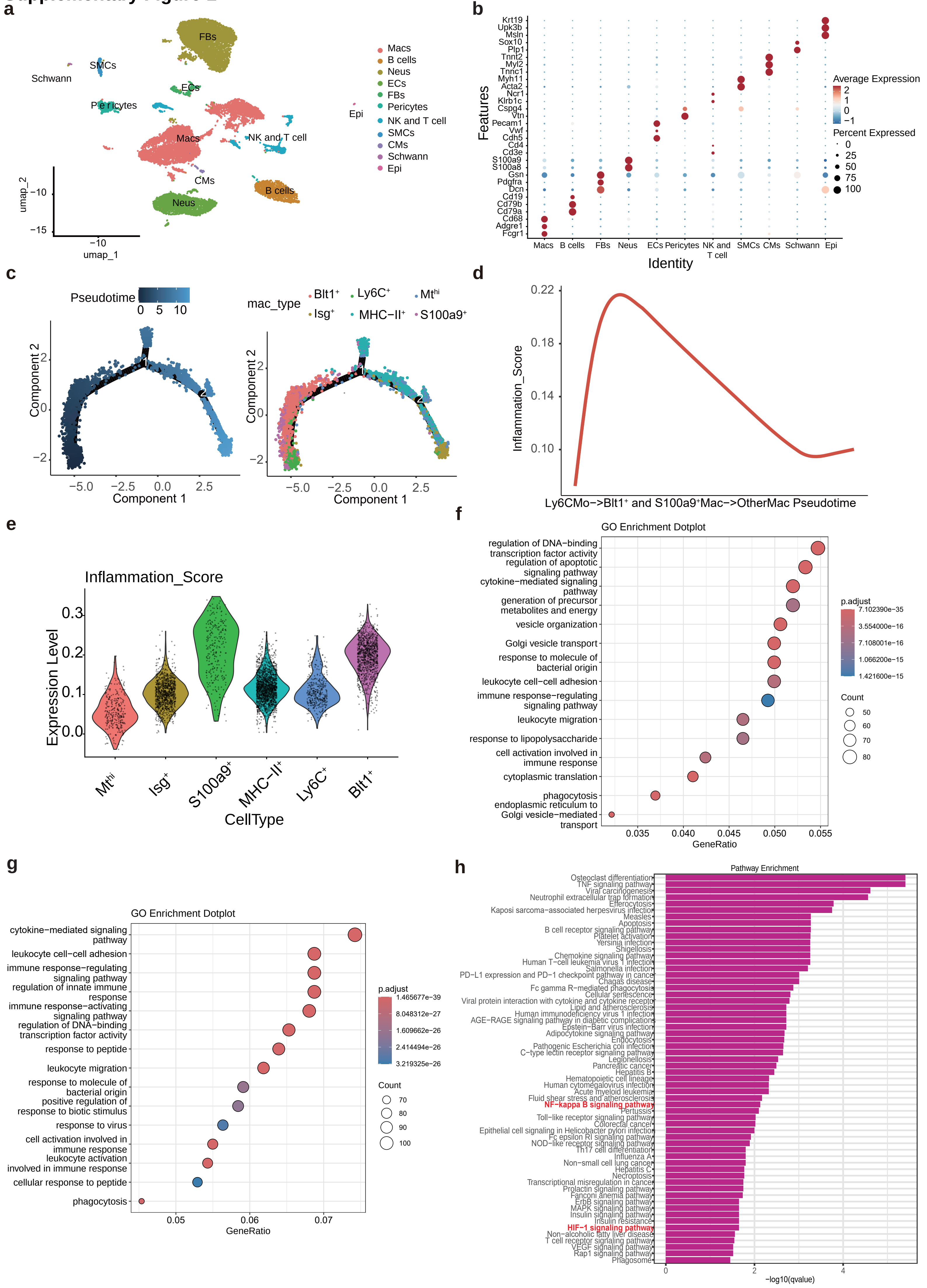

Supplementary Figure 1.

a, Identification of 11 distinct cell clusters by uniform manifold approximation and projection (UMAP) using the single-cell transcriptomics dataset GSE247139.

b, Dot plot showing the expression of marker genes in each cluster for cell-type annotation.

c, (Left) Pseudotime trajectory reconstruction of monocyte-to-macrophage differentiation. (Right) Relative abundance of each subset along pseudotime. The trajectory originated from Ly6C^{+} monocytes and branched into three terminal macrophage subsets: MHC-II^{+} macrophages, Isg^{+} macrophages and Mt^{hi} macrophages. Notably, both differentiation trajectories passed through Blt1^{+} macrophages and S100a9^{+} macrophages as intermediate states, suggesting that these populations represent transitional macrophage subsets en route to terminal differentiation.

d, Dynamic changes in the inflammation score along the pseudotime trajectory. The inflammatory score increases during the transition from Ly6C^{+} monocytes to intermediate Blt1^{+} and S100a9^{+} macrophages, followed by a decrease as cells differentiate into terminal MHC-II^{+}, Isg^{+} and Mt^{hi} macrophages.

e, Violin plot showing inflammatory scores across macrophage subsets. Blt1^{+} macrophages and S100a9^{+} macrophages exhibited significantly higher inflammatory scores than other subsets, supporting their identity as transient pro-inflammatory intermediates.

f, GO enrichment analysis of Blt1^{+} macrophages.

g, GO enrichment analysis of S100a9^{+} macrophages.

h, Top KEGG pathway analysis of DEGs in peripheral blood monocytes from patients and healthy donors.

Supplementary Figure 2

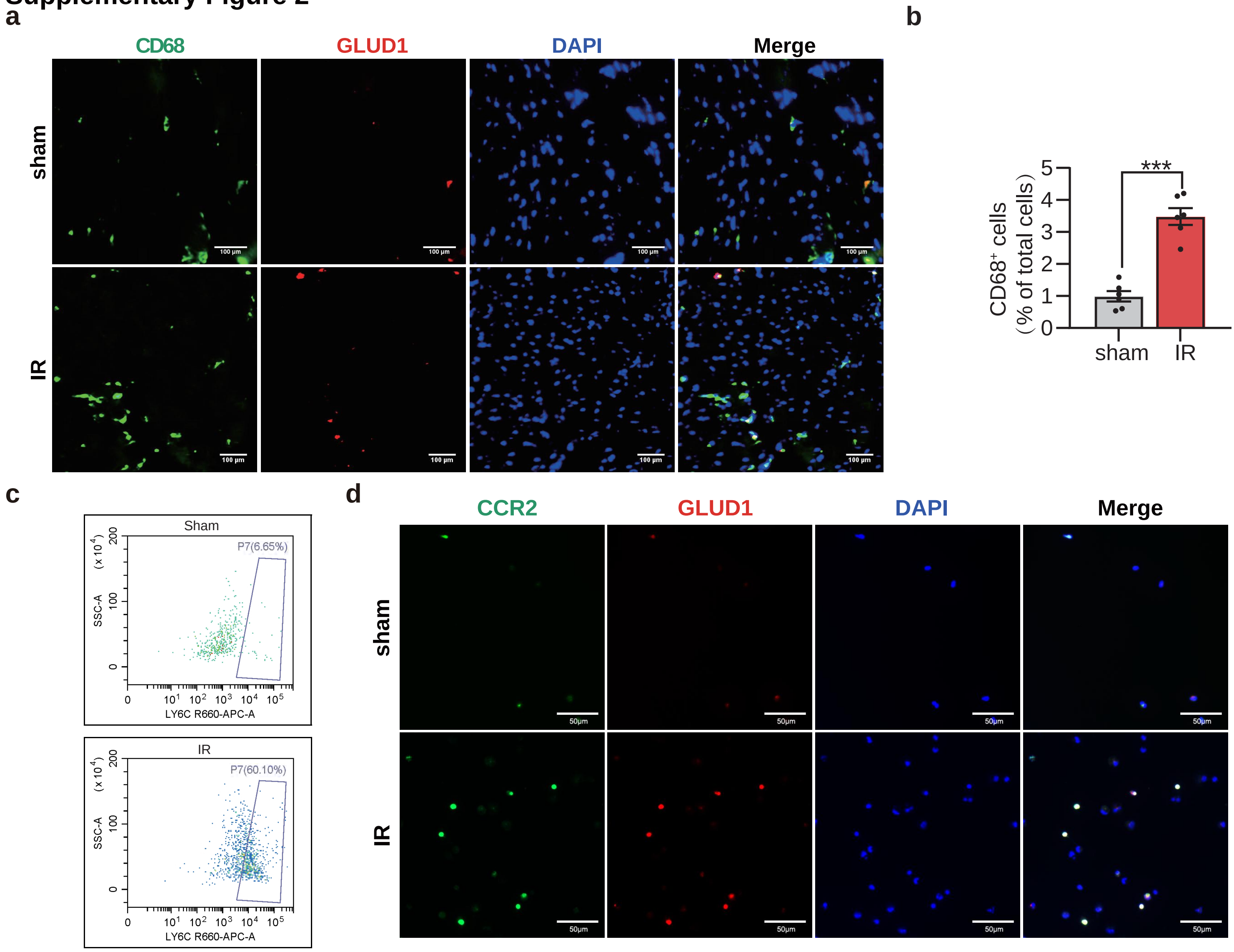

Supplementary Figure 2.

a-b, Representative images and quantification of $CD68^+$ cells in hearts from sham-operated and IR mice (n=6 per group). Scale bar, 100 µm.

c, Representative flow cytometric plots of $Ly6C^+$ cells within the cardiac $CD45^+CD11b^+F4/80^+$ monocyte/macrophage population from WT mice 24 hours post-IR injury or sham surgery.

d, Representative images and quantification of CCR2 and GLUD1 expression in cardiac $CD45^+CD11b^+F4/80^+$ cells isolated from WT mice subjected to IR injury. The percentages of $GLUD1^+$ cells within the $CCR2^+$ and $CCR2^-$ subsets were quantified (n=3 per group). Scale bar, 50 µm.

Graphs show mean ± SEM. All experiments were independently replicated at least in triplicate. Statistical analyses were conducted using two-tailed unpaired *t*-test for **b**. $^{***}P < 0.0005$.

Supplementary Figure 3

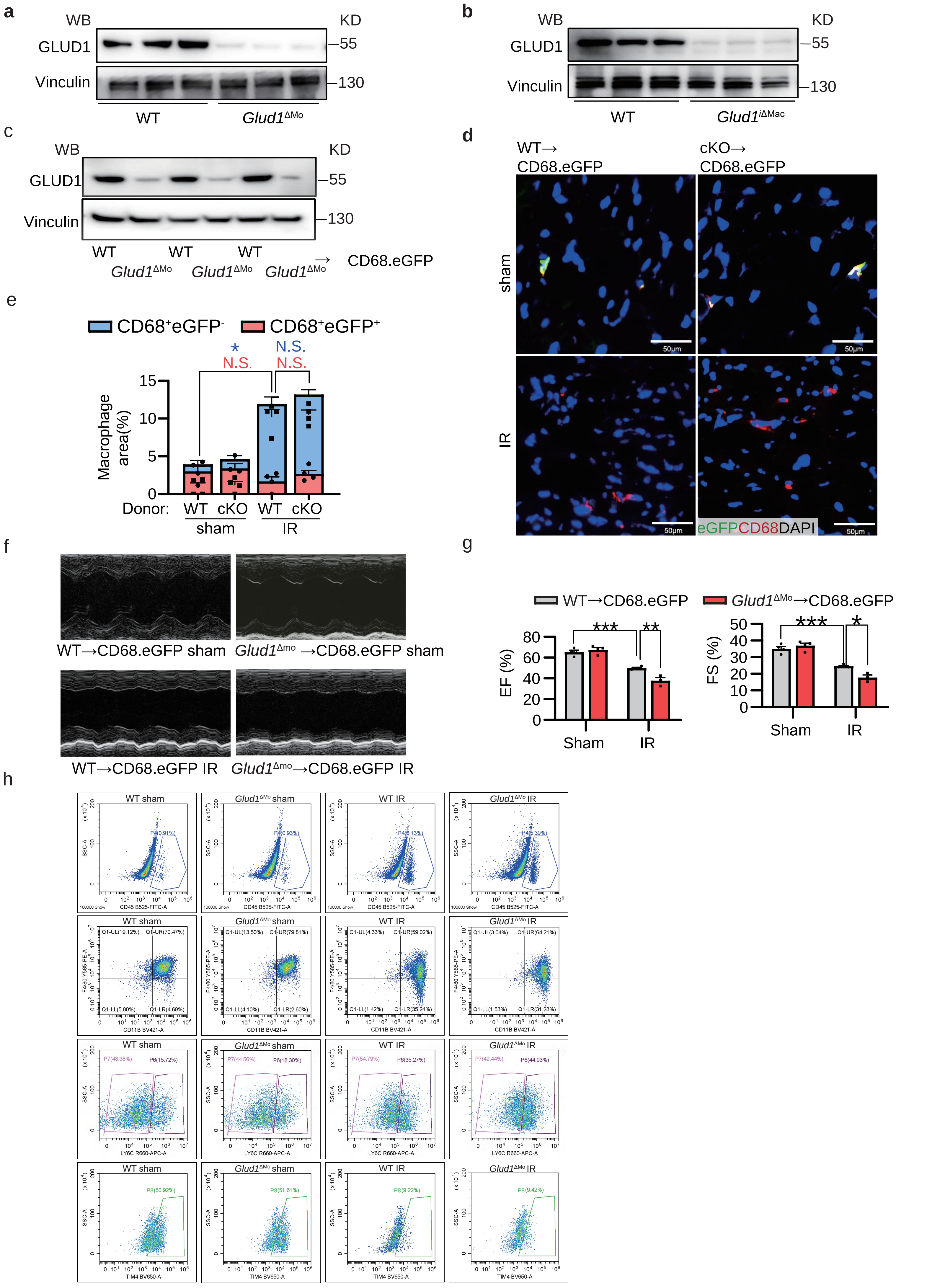

Supplementary Figure 3.

a, Western blot for GLUD1 in BMDMs from WT and *Glud1*$^{\Delta Mo}$ mice. Vinculin was used as a loading control.

b, Western blot for GLUD1 in BMDMs from WT and *Glud1*$^{i\Delta Mac}$ mice. Vinculin was used as a loading control.

c, Western blot for GLUD1 in BMDMs from WT and *Glud1*$^{\Delta Mo}$ mice transplanted into eGFP CD68 recipient mice. Vinculin was used as a loading control.

d-e, Representative images and quantification of CD68$^+$eGFP$^+$ and CD68$^+$eGFP$^-$ monocytes/macrophages in hearts from sham-operated and IR-injured BMT mice (n=4 per group). Scale bar, 50 µm.

f-g, Representative echocardiography images and quantification of ejection fraction (EF) and fractional shortening (FS) in eGFP-CD68 recipient mice transplanted with bone marrow from WT and *Glud1*$^{\Delta Mo}$ mice into 24 hours after sham surgery (n=4 per group) or IR injury (WT, n=4; *Glud1*$^{\Delta Mo}$, n=3).

h, Representative flow cytometry plots showing cardiac CD45$^+$CD11b$^+$F4/80$^+$ monocytes/macrophages, Ly6C$^+$ monocytes/macrophages, and tissue-resident TIM4$^+$Ly6C$^-$ macrophages within the CD45$^+$CD11b$^+$F4/80$^+$ monocytes/macrophages population from WT and *Glud1*$^{\Delta Mo}$ mice 24 h after IR injury. CD45$^+$CD11b$^+$F4/80$^+$ monocytes/macrophages were quantified as a percentage of total live cells using the formula: (% CD45$^+$ cells among live cells) × (% CD11b$^+$F4/80$^+$ cells among CD45$^+$ cells). Accordingly, cardiac leukocytes are displayed after gating on live CD45$^+$ cells.

Graphs show mean ± SEM. All experiments were independently replicated at least in triplicate. Statistical analyses were conducted using two-way ANOVA followed by Tukey's test for **e**,**g**. *$P < 0.05$, **$P < 0.005$, ***$P < 0.0005$, N.S., not significant ($P > 0.05$).

Supplementary Figure 4

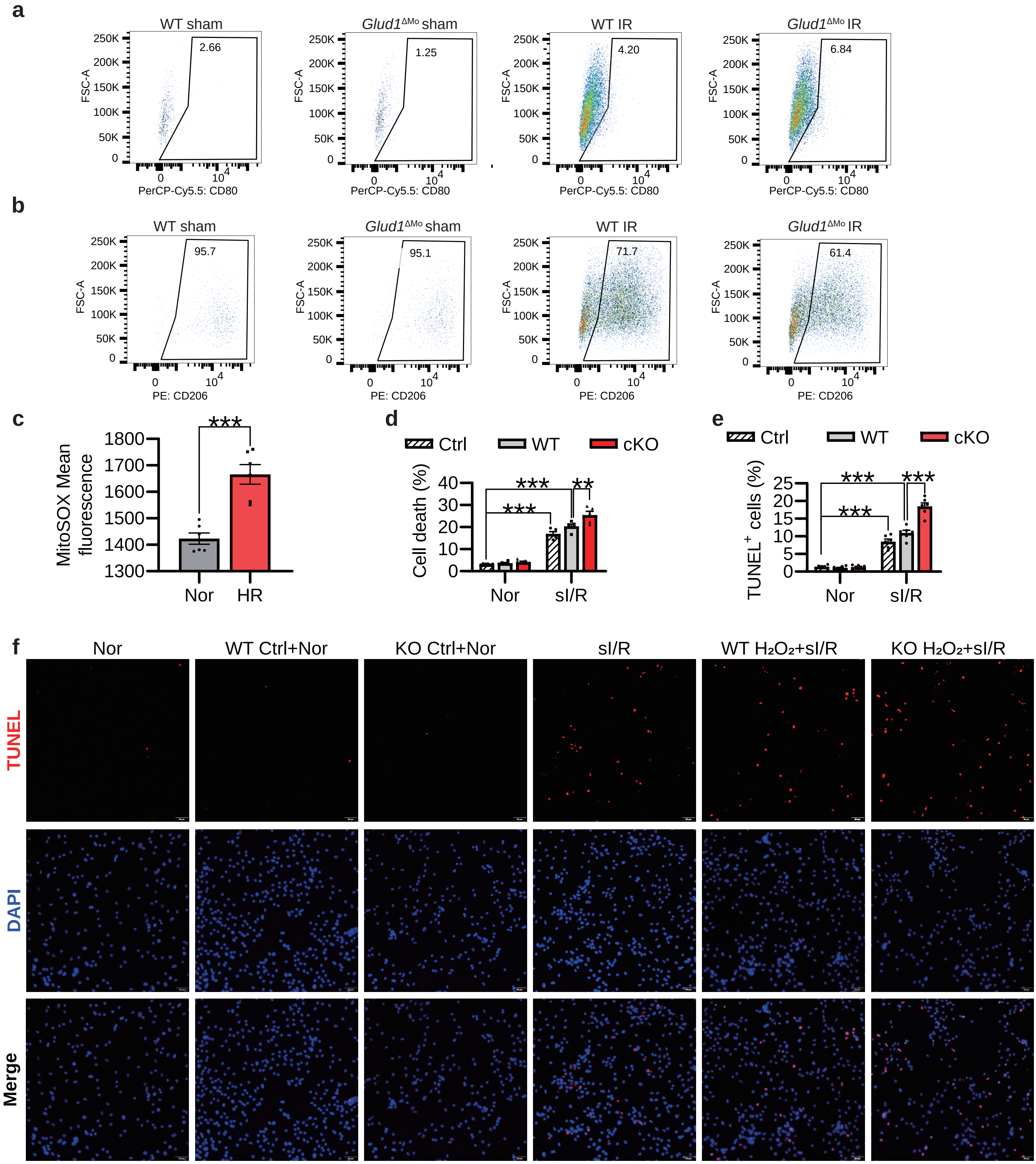

Supplementary Figure 4.

a-b, Representative flow cytometric plots of pro-inflammatory $CD80^+$ and anti-inflammatory $CD206^+$ monocytes/macrophages within $CD45^+CD11b^+CD68^+$ monocytes/macrophages from WT and *Glud1*$^{\Delta Mo}$ mice 24 hours post-IR injury.

c, Mitochondrial ROS levels in WT BMDMs assessed by flow cytometry using MitoSOX (n=6 per group).

d, Lactate dehydrogenase (LDH) levels in the NRVMs cultured with CM from H_2O_2 treated BMDMs post-sI/R treatment (Nor, n=6 per group; sI/R, n=5 per group).

e-f, Representative images and quantification of TUNEL staining in NRVMs cultured with CM from H_2O_2 treated BMDMs post-sI/R treatment (n=6 per group). Scale bar, 50 µm.

Graphs show mean ± SEM. All experiments were independently replicated at least in triplicate. Statistical analyses were conducted using two-way ANOVA followed by Tukey's test, in addition to **c**, where a two-tailed unpaired *t*-test was applied. $^{**}P < 0.005$, $^{***}P < 0.0005$.

Supplementary Figure 5

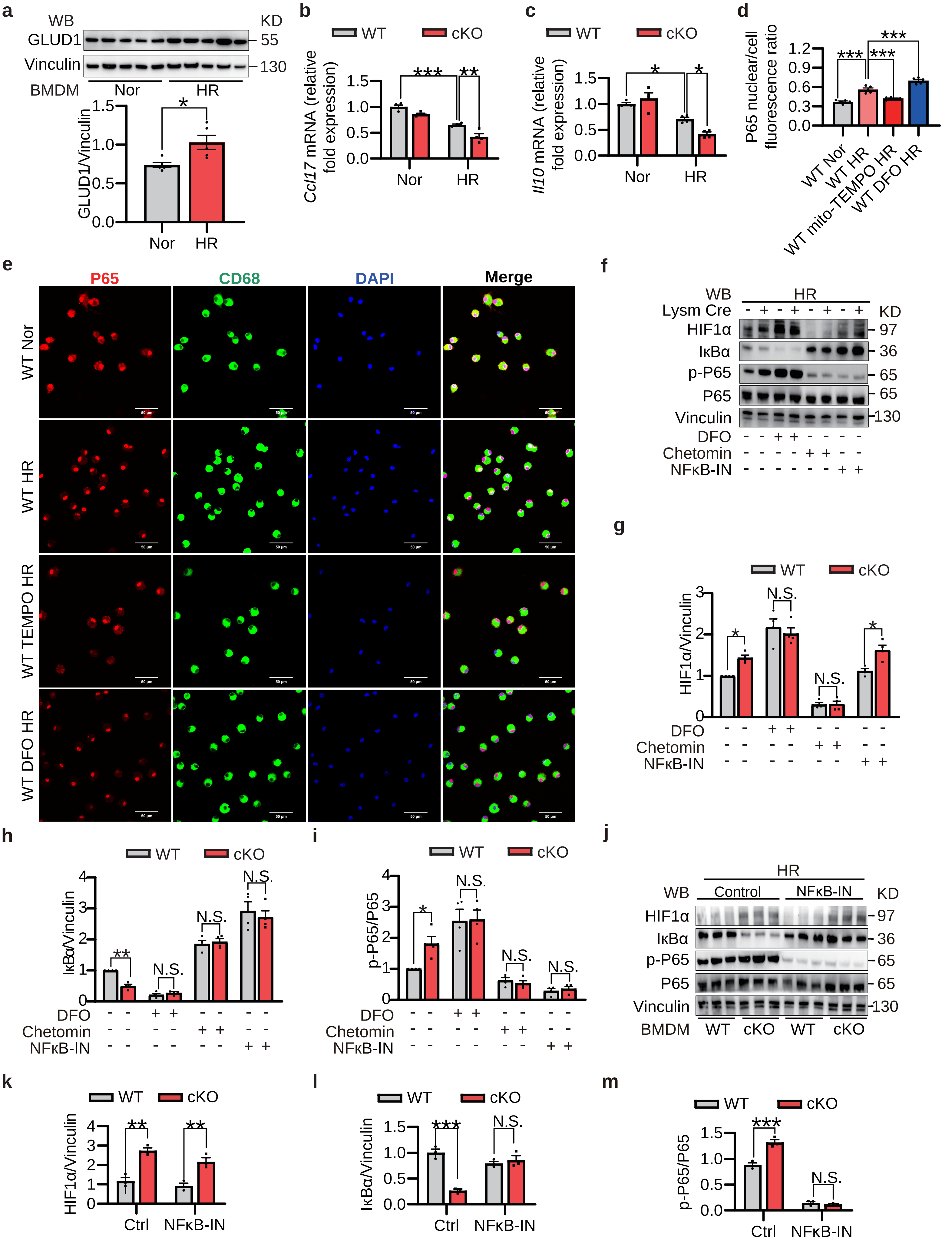

Supplementary Figure 5.

a, Western blot and quantification of GLUD1 in WT BMDM after HR stimulation. Vinculin was used as a loading control. Numbers indicate densitometric fold change relative to Vinculin (n=5 per group).

b-c, RT-qPCR analysis of anti-inflammatory genes *Ccl17* and *Il10* in BMDMs under normoxia and post-HR treatment (n=4 per group).

d-e, Representative images and quantification of P65 staining in the nucleus and cytoplasm. The ratio of P65 in the nucleus to the cytoplasm was quantified in WT BMDMs treated with MitoTEMPO (mitochondrial ROS scavenger) and DFO (PHD inhibitor) post HR treatment (n=5 per group). Scale bar, 50 μm.

f-i, Western blot and quantification of HIF1α, IκBα, p-P65, P65 in WT and cKO BMDMs treated with DFO (iron chelator and PHD inhibitor), Chetomin (HIF-1α inhibitor), and NF-κB-IN (NF-κB inhibitor) (n=4 per group) post HR treatment. Vinculin was used as a loading control. Numbers represent densitometric fold change relative to vinculin.

j-m, Western blot and quantification of HIF1α, IκBα, p-P65 and P65 in WT and cKO BMDMs treated with NF-κB-IN (NF-κB inhibitor) (n=3 per group) post HR treatment. Vinculin was used as a loading control. Numbers represent densitometric fold change relative to vinculin.

Graphs show mean ± SEM. All experiments were independently replicated at least in triplicate. Statistical analyses were conducted by two-tailed unpaired *t*-test for **a**; two-way ANOVA followed Tukey's test for **b**,**c**,**g**,**h**,**i**; one-way ANOVA for **d**; and two-way ANOVA followed by Sidak's test for **k-m**. $^{*}P < 0.05$, $^{**}P < 0.005$, $^{***}P < 0.0005$, N.S., not significant ($P > 0.05$).

Supplementary Figure 6

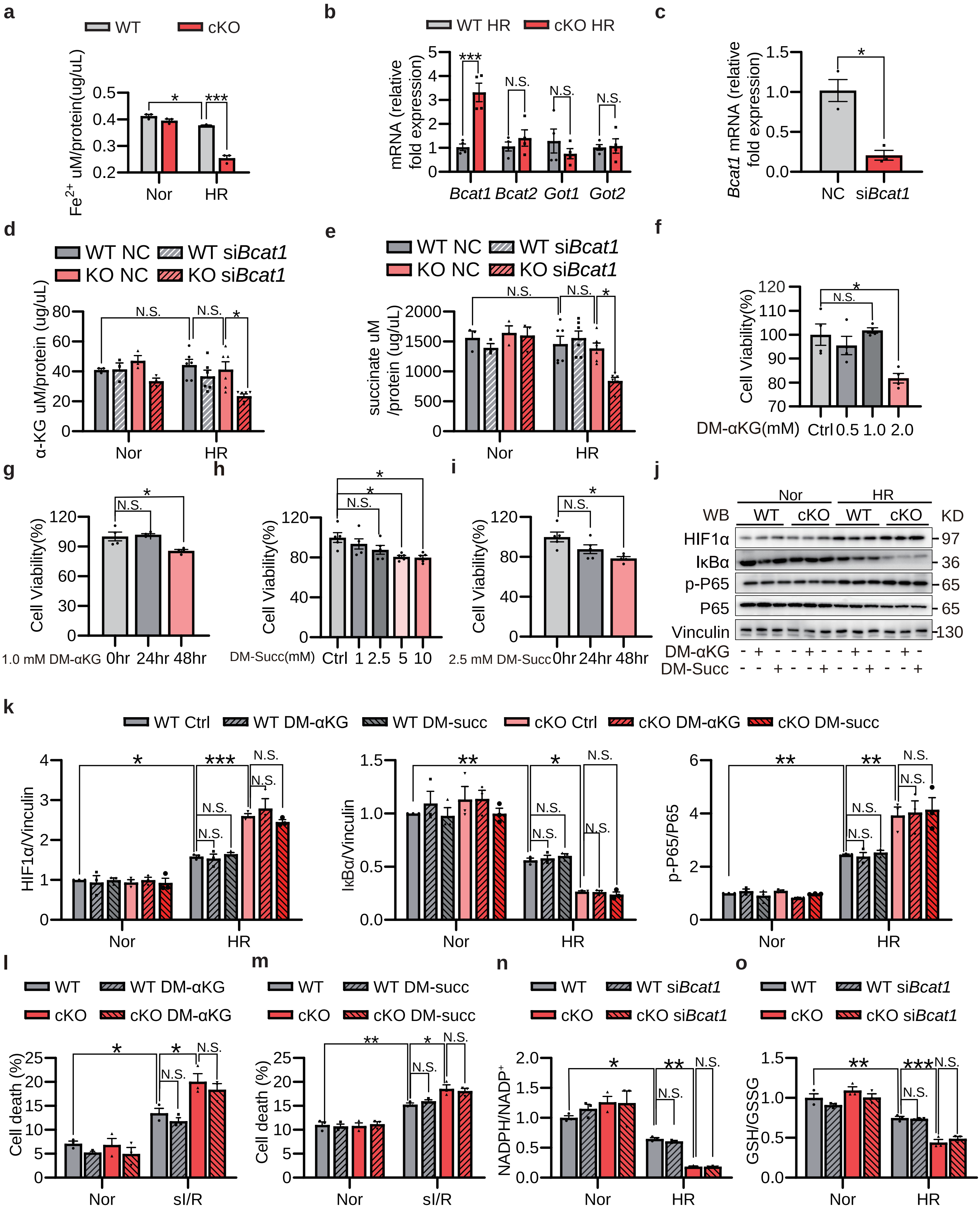

Supplementary Figure 6.

a, Measurement of Fe^{2+} levels in WT and GLUD1 KO BMDMs after HR treatment (n=3 per group).

b, RT-qPCR analysis of the aminotransferases *Bcat1*, *Bcat2*, *Got1* and *Got2* in WT and GLUD1 KO BMDMs after HR treatment (n=4 per group).

c, RT-qPCR analysis of *Bcat1* expression to evaluate the efficiency of *Bcat1* silencing (n=3 per group).

d-e, Measurement of α-KG and succinate levels using commercial assay kits in WT and GLUD1 KO BMDMs upon *Bcat1* silencing (Nor, n=3 per group; HR, n=6 per group).

f-g, BMDMs were treated with the indicated concentrations of α-ketoglutarate (DM-αKG) for 24 h (**f**) or with 1 mM DM-αKG for 24 or 48 h (**g**), and cell viability was assessed using the CCK-8 assay (n = 4 per group).

h-i, BMDMs were treated with the indicated concentrations of dimethyl succinate (DM-succ) for 24 h (**h**) or with 2.5 mM DM-succ for 24 or 48 h (**i**), and cell viability was assessed using the CCK-8 assay (n = 5 per group).

j-k, Western blot and quantification of HIF1α, IκBα, p-P65 and P65 in WT and GLUD1 KO BMDMs treated with DM-αKG or DM-succ after HR treatment (n=3 per group). Vinculin was used as a loading control. Numbers represent densitometric fold change relative to vinculin.

l-m, Cell viability assay in NRVMs subjected to sI/R injury following DM-αKG or DM-succ treatment (n=3 per group).

n-o, Measurement of NADPH/$NADP^+$ and GSH/GSSG ratios using commercial kits in WT and GLUD1 KO BMDMs upon *Bcat1* silencing (n=3 per group).

Graphs show mean ± SEM. All experiments were independently replicated at least in triplicate. Statistical analyses were conducted using two-way ANOVA followed by Tukey's test for **a**,**d**,**e**,**k-o**, two-way ANOVA followed by Sidak's test for **b**, two-tailed unpaired *t*-test for **c**, and one-way ANOVA for **f-i**. $*P < 0.05$, $**P < 0.005$, $***P < 0.0005$, N.S., not significant ($P > 0.05$).

Supplementary Figure 7

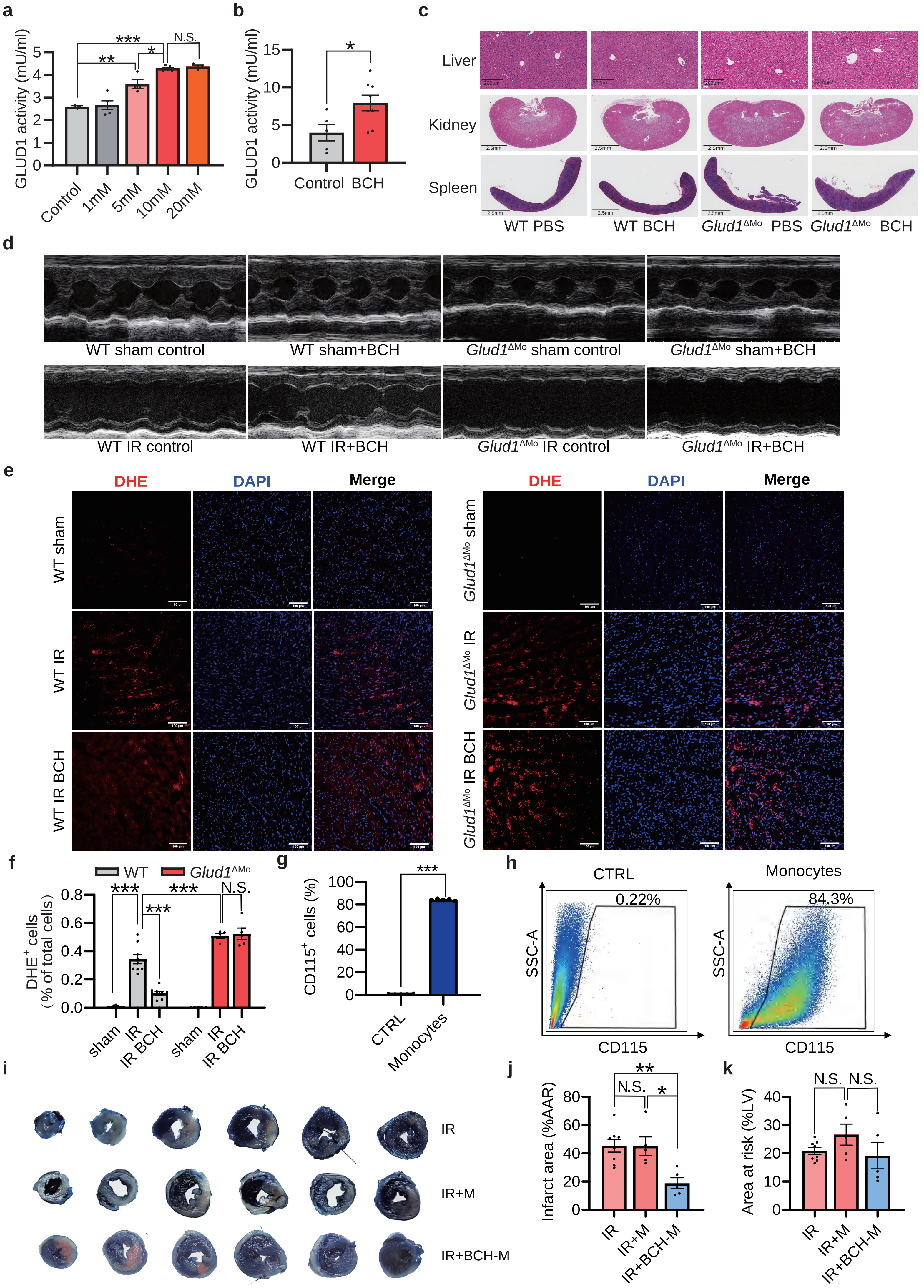

Supplementary Figure 7.

a, GLUD1 activity was assessed in BMDMs treated with a range of BCH concentrations for 24 hours (Ctrl, n=3; 1 mM, 10 mM, n=5; 5 mM, 20 mM, n=4).

b, GLUD1 activity was assessed in peritoneal macrophages isolated from mice treated with 0.7 mg/g body weight of BCH (control, n=5; BCH, n=8).

c, Representative images of hematoxylin and eosin (HE) staining on the liver, kidney, and spleen in BCH-treated WT and *Glud1*$^{\Delta Mo}$ mice. Scale bars, 200 μm (liver) and 2.5 mm (kidney and spleen).

d, Representative echocardiography images of EF and FS in WT and *Glud1*$^{\Delta Mo}$ mice treated with BCH or PBS post-sham or IR injury (n=6 per group).

e-f, Representative images and quantification of DHE-positive cells in heart tissues of BCH-treated WT and *Glud1*$^{\Delta Mo}$ mice (WT group, n=9; *Glud1*$^{\Delta Mo}$ group, n=5). Scale bar, 100 μm.

g-h, Monocytes were purified from peripheral blood mononuclear cells (PBMCs) using CD115 magnetic beads, and their purity was verified by flow cytometric analysis with an anti-CD115 antibody (n=5 per group).

i-k, LV tissue sections from mice transfused with BCH-treated monocytes (IR+BCH-M) or PBS-treated monocytes (IR+M) were stained with Evans Blue/TTC to evaluate the myocardial infarction area after IR. AAR and the IF/AAR ratio were quantified (IR, n=8; IR+M, n=5; IR+BCH-M, n=5).

Graphs show mean ± SEM. All experiments were independently replicated at least in triplicate. Statistical analyses were conducted using one-way ANOVA followed by Tukey's test in addition to **b**,**g**, where two-tailed unpaired *t*-test was applied, and **f**, where one-way ANOVA followed by Tukey's test was applied. *$P < 0.05$, **$P < 0.005$, ***$P < 0.0005$, N.S., not significant ($P > 0.05$).
