# Supplementary material for: Macrophage GLUD1 is Essential for Redox Balance and Immune Homeostasis in Cardiac Injury
Source: Adv Sci (Weinh). 2026 Oct 28:e78416. Online ahead of print. doi: 10.1002/advs.78416 (PMC13717128; doi:10.1002/advs.78416)
Supplement: Supplementary file 2 [file ADVS-9999-e78416-s002.pdf]

Table 1. Echocardiographic measurements for *Glud1*^{ΔMo} and WT mice at 24 h after IR injury.

Parameter	WT sham (n=6)	*Glud1*^{ΔMo} sham (n=7)	WT IR (n=10)	*Glud1*^{ΔMo} IR (n=8)
LVPWs (mm)	1.41 ± 0.25	1.28 ± 0.31	1.40 ± 0.52	0.93 ± 0.14
LVIDd (mm)	3.37 ± 0.21	3.67 ± 0.47	3.86 ± 0.37	3.88 ± 0.59
LVIDs (mm)	2.07 ± 0.31	2.26 ± 0.42	2.73 ± 0.27$^{\#}$	3.06 ± 0.58*
LVESV (µl)	14.37 ± 5.45	18.34 ± 7.65	28.25 ± 6.88$^{\#}$	38.65 ± 19.11*
FS (%)	38.72 ± 6.89	38.69 ± 5.49	29.20 ± 2.39$^{\#}$	21.40 ± 4.07*
EF (%)	69.70 ± 8.26	69.56 ± 6.82	56.71 ± 3.63$^{\#}$	43.89 ± 7.52*

LVPWs, left ventricular posterior wall thickness; LVIDd, left ventricular end-diastolic dimension; LVIDs, left ventricular end- systolic dimension; LVESV, left ventricular end-systolic volume; FS, fractional shortening; EF, ejection fraction. Graphs show mean ± SEM. $^{\#}P < 0.05$ versus WT sham group; $^{*}P < 0.05$ versus WT IR group.

Table 2. Echocardiographic measurements in CD68 eGFP mice reconstituted with WT or *Glud1*^{Mo} bone marrow 24 h after IR injury.

Parameter	WT into eGFP CD68 Sham (n=4)	*Glud1*^{Mo} into eGFP CD68 Sham (n=4)	WT into eGFP CD68 IR (n=4)	*Glud1*^{Mo} into eGFP CD68 IR (n=3)
LVPWs (mm)	1.45 ± 0.16	1.34 ± 0.13	1.29 ± 0.18	0.82 ± 0.17*
LVIDd (mm)	3.57 ± 0.07	3.83 ± 0.24	3.56 ± 0.35	3.64 ± 0.25
LVIDs (mm)	2.32 ± 0.10	2.42 ± 0.25	2.68 ± 0.27	2.99 ± 0.24
LVESV (µl)	18.59± 2.05	20.85 ± 5.78	26.85 ± 6.86	35.04 ± 6.94
FS (%)	35.06 ± 2.67	37.03 ±2.70	24.65 ± 0.91$^{\#}$	17.86 ± 2.59*
EF (%)	65.27 ± 3.59	67.59 ±3.74	49.85 ± 1.53$^{\#}$	37.84 ± 4.89*

LVPW, left ventricular posterior wall thickness; LVIDd, left ventricular end-diastolic dimension; LVIDs, left ventricular end- systolic dimension; LVESV, left ventricular end-systolic volume; FS, fractional shortening; EF, ejection fraction. Graphs show mean ± SEM. $^{\#}P < 0.05$ versus WT sham group; $^{*}P < 0.05$ versus WT IR group.

Table 3. Echocardiographic measurements for BCH-or vehicle treated mice at 24h after IR injury.

Parameter	Vehicle WT sham (n=6)	BCH treated WT sham (n=6)	Vehicle *Glud1*$^{\Delta Mo}$ sham (n=6)	BCH treated *Glud1*$^{\Delta Mo}$ sham (n=6)	Vehicle WT IR (n=6)	BCH treated WT IR (n=6)	Vehicle *Glud1*$^{\Delta Mo}$ IR (n=6)	BCH treated *Glud1*$^{\Delta Mo}$ IR (n=6)
LVPWs (mm)	1.66 ± 0.16	1.68 ± 0.14	1.74 ± 0.15	1.65 ± 0.10	0.98 ± 0.18$^{\$}$	1.16 ± 0.11	1.03 ± 0.33	1.01 ± 0.23
LVIDd (mm)	2.49 ± 0.21	2.30 ± 0.25	2.41 ± 0.16	2.13 ± 0.47	3.98 ± 0.45$^{\$}$	3.72 ± 0.57	4.04 ± 0.40	3.80 ± 0.39
LVIDs (mm)	1.01 ± 0.19	0.86 ± 0.16	0.91 ± 0.10	1.24 ± 0.85	2.91 ± 0.33$^{\$}$	2.46 ± 0.36	3.21 ± 0.40	3.01 ± 0.37
LVESV (µl)	2.33 ± 1.01	1.51 ± 0.65	1.67 ± 0.47	1.88 ± 0.85	33.04 ± 8.61$^{\$}$	22.23 ±6.78	41.11 ± 13.28	35.41 ± 11.26
FS (%)	59.60 ± 4.92	62.62 ± 5.58	62.41 ± 3.16	61.17 ± 1.56	26.85 ± 0.45$^{\$}$	33.65 ± 0.78$^{\#}$	20.65 ± 2.51*	20.90 ± 2.66
EF (%)	89.86 ± 3.06	91.88 ± 3.23	91.87 ± 1.78	91.39 ± 0.99	52.96 ± 0.65$^{\$}$	63.21 ± 0.55$^{\#}$	42.55 ± 4.66*	43.15 ± 4.83

LVPW, left ventricular posterior wall thickness; LVIDd, left ventricular end-diastolic dimension; LVIDs, left ventricular end- systolic dimension; LVESV, left ventricular end-systolic volume; FS, fractional shortening; EF, ejection fraction. Graphs show mean ± SEM. $^{\$}$*P* < 0.05 versus Vehicle WT Sham group; $^{\#}$*P* < 0.05 versus Vehicle WT IR group; **P* < 0.05 versus VehicleWT IR group.

Table 4. Echocardiographic measurements for mice that underwent transfusion of BCH-treated monocytes.

Parameter	Sham (n=5)	Sham + M (n=5)	IR (n=5)	IR + M (n=7)	IR + BCH-M (n=7)
LVPWs (mm)	1.38 ± 0.10	1.45 ± 0.12	0.84 ± 0.23$^{\$}$	1.03 ± 0.25$^{\#}$	1.09 ± 0.15
LVIDd (mm)	3.42 ± 0.39	3.52 ± 0.32	3.84 ± 0.27	3.82 ± 0.21	3.39 ± 0.20
LVIDs (mm)	2.03 ± 0.24	2.11 ± 0.20	3.03 ± 0.40$^{\$}$	3.10 ± 0.28$^{\#}$	2.39 ± 0.18*
LVESV (µl)	13.50 ± 4.10	14.81 ± 3.36	36.56 ± 12.78$^{\$}$	38.51 ± 8.51$^{\#}$	20.10 ± 3.56*
FS (%)	40.65 ± 2.12	40.09 ± 0.93	21.46 ± 5.04$^{\$}$	18.73 ± 3.60$^{\#}$	29.52 ± 3.49*
EF (%)	72.50 ± 2.48	71.69 ± 1.20	43.88 ± 9.11$^{\$}$	39.18 ± 6.49$^{\#}$	57.50 ± 5.38*

LVPW, left ventricular posterior wall thickness; LVIDd, left ventricular end-diastolic dimension; LVIDs, left ventricular end-systolic dimension; LVESV, left ventricular end-systolic volume; FS, fractional shortening; EF, ejection fraction. Graphs show mean ± SEM. $^{\$}P < 0.05$ versus Sham group; $^{\#}P < 0.05$ versus Sham+M group; $^{*}P < 0.05$ versus IR+M group.
