# Supplementary material for: Macrophage GLUD1 is Essential for Redox Balance and Immune Homeostasis in Cardiac Injury
Source: Adv Sci (Weinh). 2026 Oct 28:e78416. Online ahead of print. doi: 10.1002/advs.78416 (PMC13717128; doi:10.1002/advs.78416)
Supplement: Supplementary file 3 [file ADVS-9999-e78416-s004.pdf]

Appendix 1

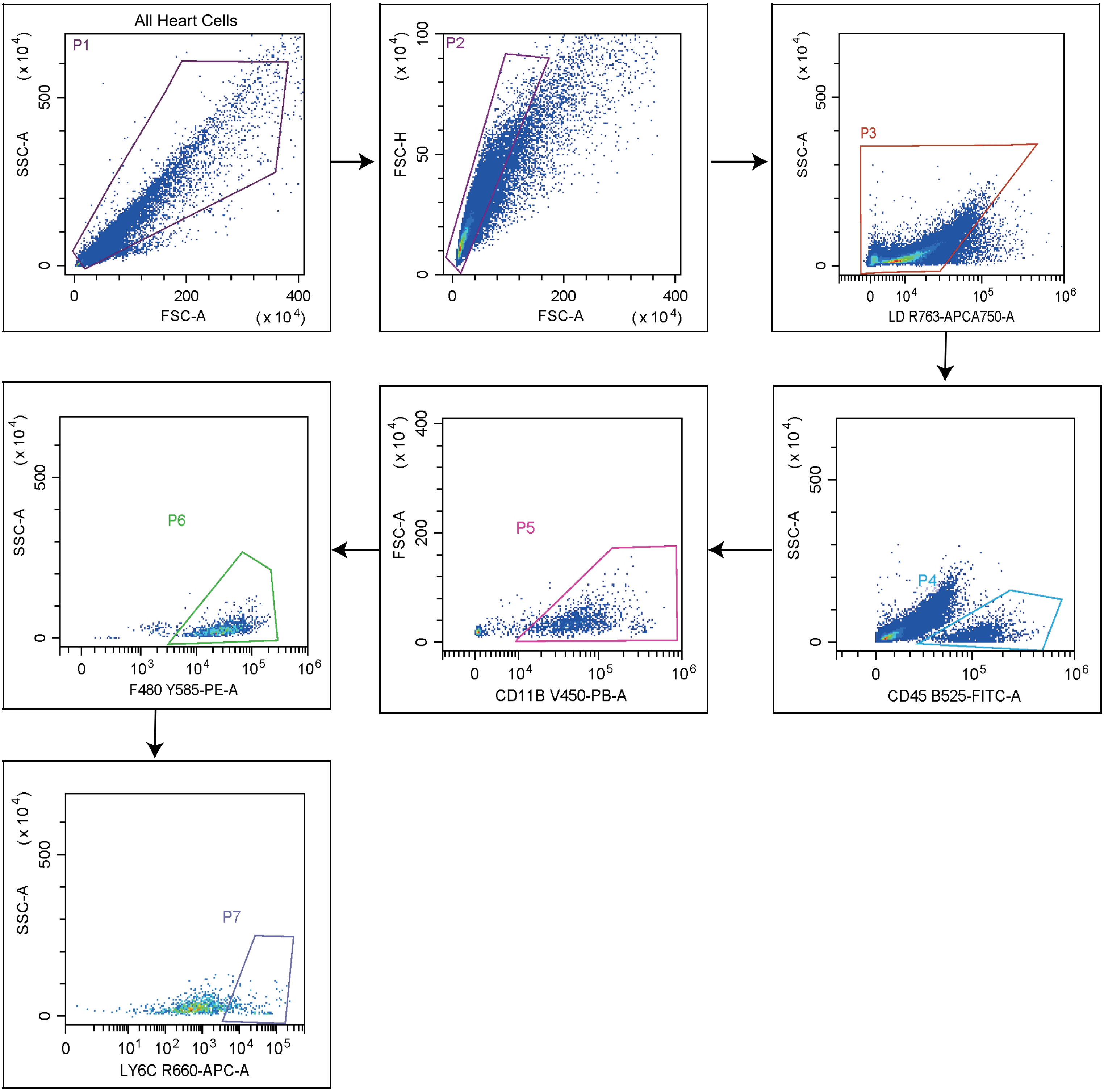

The gating strategy used for flow cytometry analysis: Live cells (APC-Cy7^{Lo}) were selected based on FSC/SSC. Gating on leukocytes based on CD45, Ly6c^{+}/CD11b^{+}/F4/80^{+} dentified pro-inflammatory macrophages.

Appendix 2

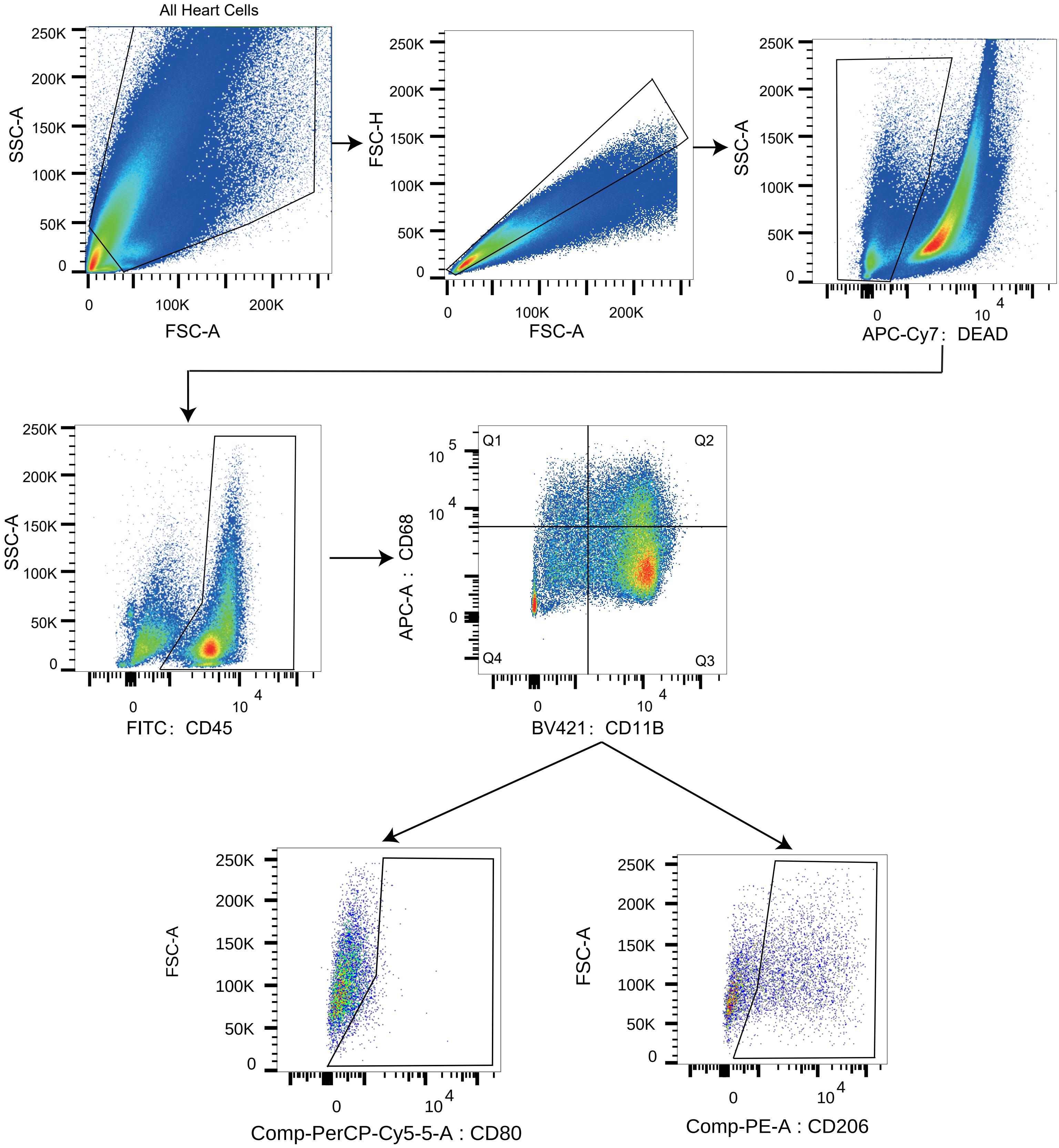

The gating strategy used for flow cytometry analysis: Live cells (APC-Cy7^{Lo}) were selected based on FSC/SSC. Gating on leukocytes based on CD45, CD68^{hi}/CD11b^{hi} identified macrophages. CD206^{hi} macrophages correspond to CD80^{hi} macrophages.

Appendix 3

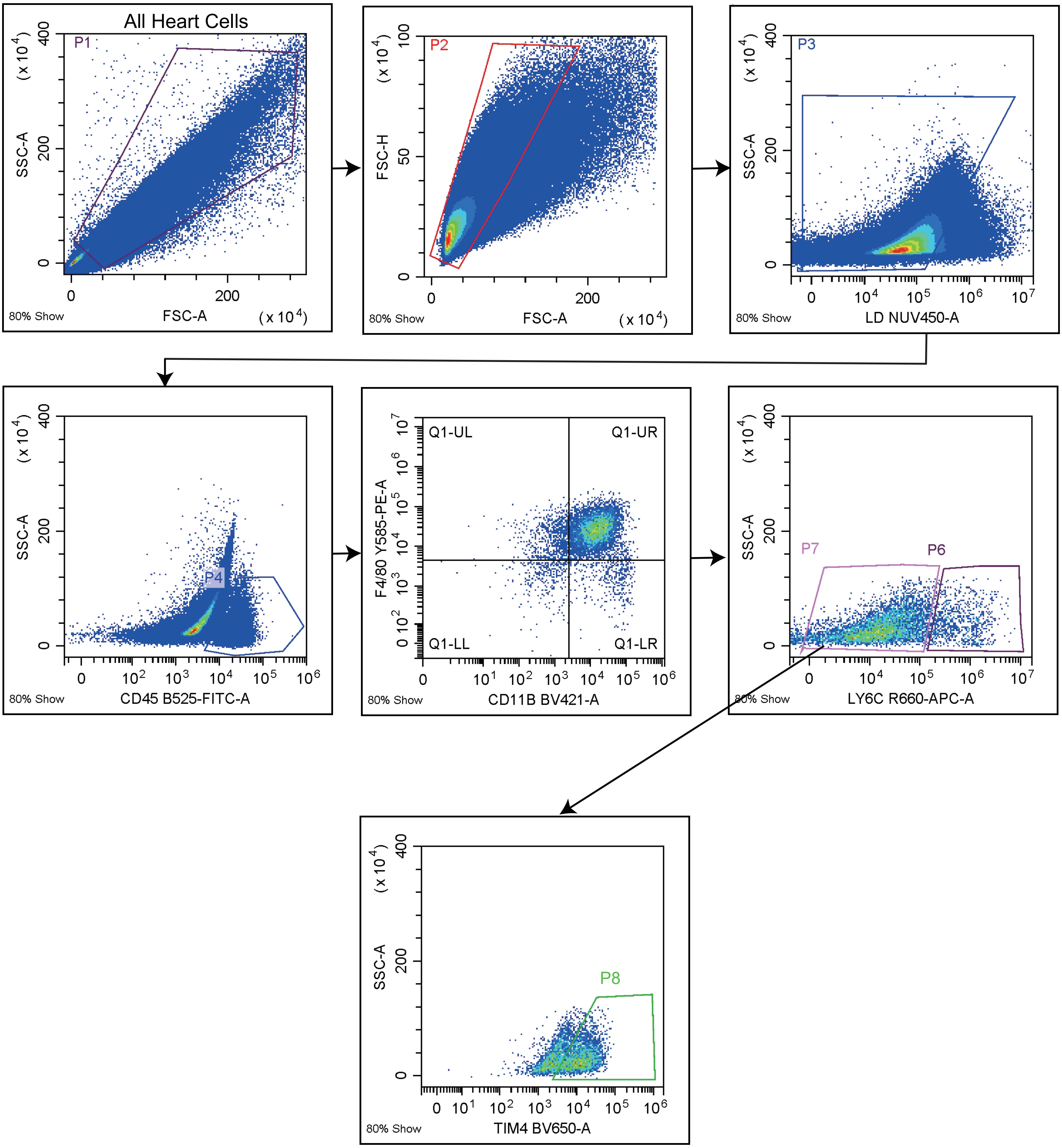

The gating strategy used for flow cytometry analysis: Live cells (BUV395^Lo) were selected based on FSC/SSC. Gating on leukocytes based on CD45, Ly6C⁺/CD11b⁺/F4/80⁺ dentified infiltrating pro-inflammatory macrophages, Ly6C⁻/Tim4⁺/CD11b⁺/F4/80⁺ dentified tissue-resident macrophages.
