# Supplementary material for: Macrophage GLUD1 is Essential for Redox Balance and Immune Homeostasis in Cardiac Injury
Source: Adv Sci (Weinh). 2026 Oct 28:e78416. Online ahead of print. doi: 10.1002/advs.78416 (PMC13717128; doi:10.1002/advs.78416)
Supplement: Supplementary file 4 [file ADVS-9999-e78416-s003.docx]

**Supplemental Material**

**Methods**

**Animals:** Floxed *Glud1* (*Glud1^tm1.1Pma^*, MGI:3835667)^1^ on a C57BL/6 background were intercrossed. *Glud1*^L/L^ x CSF1R:Cre-ERT transgenic mice were generated by intercrossing *Glud1* floxed mice with the tamoxifen-inducible CSF1R:Cre-ERT deleter mouse line^2^, which mediates recombination in monocytes and macrophages. Similarly, *Glud1*^L/L^ x LysM:Cre mice were generated by intercrossing *Glud1* floxed mice with a myeloid cell delete LysM:Cre transgenic mice. Both conditional lines have been described and characterized previously^3^. The strains were bred and transferred from the lab of Dr. Massimiliano Mazzone (University of Leuven, Belgium). Male mice aged 8 to 12 weeks with homozygous *Glud1* conditional knockout were used as the GLUD1-cKO group, while male wild-type (WT) littermates were used as the controls. To induce the deletion of *Glud1* in monocytes and macrophages, tamoxifen (0.05 mg/g body weight) was administered daily by intraperitoneal (i.p.) injection for 5 consecutive days in *Glud1*^L/L^ x CSF1R:Cre-ERT mice. *Glud1*^L/L^ x CSF1R:Cre-negative mice that received tamoxifen following the same protocol were used as controls. All animal experiments were approved by the Animal Care and Use Committee of the School of Medicine, Zhejiang University.

**Busulfan-based bone marrow transplantation:** Busulfan (5 mg; Merck, B2635) was weighed and dissolved in 0.5 mL sterile dimethyl sulfoxide (DMSO) with vigorous shaking until fully dissolved. Subsequently, 0.5 mL of the resulting solution was added to 4.5 mL Ca^2+^/Mg^2+^-free PBS prewarmed to 37°C to yield a final Busulfan concentration of 1 mg/mL. The solution was sterilized by filtration through a 0.2 μm syringe filter. Each mouse (20 g) received 0.4 mL of the Busulfan solution by intraperitoneal injection. The same procedure was repeated 24 hours later. Twenty-four hours after the second injection, donor bone marrow cells were isolated under sterile conditions, and 2 × 10^6^ bone marrow cells were injected intravenously into each recipient mouse via the tail vein. Mice were allowed to recover for 6 weeks to ensure adequate hematopoietic reconstitution.

**Echocardiography:** Transthoracic echocardiography was performed using a Vevo 3100 imaging system (Visual Sonics, MX400 probe) equipped with a 30 MHz imaging transducer. Mice were anesthetized with 2% isoflurane gas for echocardiographic assessment, conducted both before ischemia/reperfusion (IR) and 24 hours after IR. Body temperature was stabilized using a heating pad, and heart rate was maintained at approximately 500 beats per minute, as previously described^4^. M-mode images were acquired at the level of the left ventricular papillary muscles. The left ventricular end-diastolic internal dimension (LVIDd), left ventricular end-systolic internal dimension (LVIDs), left ventricular end-diastolic volume (LVEDV), left ventricular end-systolic volume (LVESV), and left ventricular posterior wall thickness at end-systole (LVPWs) were measured. Fractional shortening was measured from the recordings. Fractional shortening (FS%) was calculated using the formula: FS% = [(LVIDd-LVIDs)/LVIDd] × 100%. Left ventricular ejection fraction (LVEF%) was calculated as: LVEF (%) = [(LVEDV - LVESV) / LVEDV] × 100%.

**Cardiac ischemia reperfusion injury model:** Male mice aged 10–12 weeks were subjected to cardiac ischemia reperfusion (IR) surgery, as described previously^5^. In brief, mice were anesthetized with isoflurane and maintained on a heating pad to preserve body temperature. After intubation with an 18‐G tracheal tube, the mice were ventilated using a MiniVent mouse ventilator. A left thoracotomy was performed to expose the left anterior descending (LAD) coronary artery, which was ligated 2 mm below the tip of the left atrium using a 6-0 silk suture. After 45 minutes of coronary occlusion, the ligation was released to restore blood flow. Sham operated mice underwent thoracotomy and closure without LAD ligation.

**Infarct size assessment:** After 24 hours of reperfusion, the mice were anesthetized, the LAD artery was re-ligated at the original ligation site. An insulin needle was inserted under the three branches of the aortic arch toward the apex of the heart, and 0.3 mL of 1% (w/v) Evans Blue dye was injected. The non-ischemic myocardium was stained blue, while the remaining myocardium would be the ischemic risk area. The heart was quickly excised, washed, snap-frozen, and sliced into transverse sections approximately 1 mm thick. The slices were incubated in 2% TTC solution at 37°C for 30 minutes. The infarcted area was stained grayish-white, while the viable myocardium within the AAR was stained red. After rinsing with PBS, it was fixed in 10% paraformaldehyde and photographed. Infarct size and AAR were quantified using ImageJ software, and relative ratios were calculated.

**BCH treatment:** For *in vivo* experiments, BCH was administered i.p. at a dose of 0.7 mg/g body weight. The solution was prepared by dissolving BCH in sterile PBS. After 45 minutes of LAD ligation, the blood flow was restored, and BCH was i.p. injected. The control group received an equal volume of PBS. Cardiac function was evaluated by echocardiography 24 hours after reperfusion. For *in vitro* experiments, bone marrow-derived macrophages (BMDMs) were treated with BCH at a concentration determined through a concentration gradient experiment.

**Adoptive transfer of peripheral blood-derived monocytes:** Peripheral blood was collected by terminal retro-orbital exsanguination from 10-12-week-old male C57BL/6 mice into heparinized tubes, yielding approximately 0.8-1 mL per donor. Blood from five donors was pooled for each recipient mouse to ensure sufficient cell yield. As previously described^6,7^, peripheral blood mononuclear cells (PBMCs) were isolated from pooled blood using Ficoll-Paque™ Plus (YEASEN, 40504ES60) density-gradient centrifugation. In brief, pooled blood was diluted 1:1 with PBS, and layered over 3 mL of Ficoll-Paque™ Plus. Samples were centrifuged at 400 x g for 30 min at room temperature. The PBMC layer at the plasma-Ficoll interface was carefully collected, washed twice with PBS, and resuspended in Dulbecco’s modified Eagle’s medium (DMEM) supplemented with 5% fetal bovine serum (FBS) and 1% penicillin-streptomycin.

Monocytes were subsequently enriched from the PBMC fraction using CD115 magnetic beads (Miltenyi Biotec, 30096354, Germany) according to the manufacturer's instructions. Flow cytometric analysis confirmed that approximately 85% of the enriched cells were CD115⁺. The enriched monocytes were treated with 10 mM BCH or an equal volume of the vehicle control for 24 h. Subsequently, BCH-treated or vehicle-treated monocytes were collected, washed with PBS, and resuspended in sterile PBS. Each recipient mouse received 1 × 10^6^ enriched cells by tail vein injection. Cell numbers were determined by direct counting immediately before injection. Five groups were included: sham, sham with vehicle-treated monocytes, IR, IR with vehicle-treated monocytes, and IR with BCH-treated monocytes. Mice in the sham and IR groups received an equal volume of sterile PBS without cells.

**Analysis of cardiac tissue macrophages by flow cytometry:** Hearts were perfused with phosphate-buffered saline (PBS) to remove blood, minced, and digested in cardiac digestion solution, containing 2 mg/ml collagenase IV, 1 mg/ml dispase II, and 0.2 mg/ml DNase in Hanks’ balanced salt solution (HBSS) at 37°C for 60 minutes with gentle agitation at 130 rpm. The solution was subsequently filtered through a 40 μm cell strainer in a 50 mL centrifuge tube, and centrifuged at 1100 rpm for 10 minutes. Cells were suspended in red blood cell lysis buffer, and incubated for 5 minutes. Afterwards, cells were incubated with Block Fc receptors (anti-CD16/CD32) at 4°C for 5 minutes, followed by staining with anti-CD45 (BD Biosciences, FAB114G-100UG, 1:100), anti-CD11b (BioLegend, 101236, 1:100), anti-CD68 (BioLegend, 137008, 1:100) or anti-F4/80 (BioLegend, 111604, 1:100), anti-Ly6C (BioLegend, 128016, 1:100), anti-TIM4 (BD Biosciences, 745363, 1:100), anti-CD80 (BioLegend, 104712, 1:100) and anti-CD206 (BioLegend, 141706, 1:100) at 4°C for 30 minutes. After incubation, cells were washed, resuspended in cold FACS buffer, and analyzed by flow cytometry.

**Flow cytometry sorting of cardiac macrophages:** For sorting experiments, cardiac single-cell suspensions were prepared as described above and stained with anti-CD45 (BD Biosciences, FAB114G-100UG, 1:100), anti-CD11b (BioLegend, 101236, 1:100), anti-F4/80 (BioLegend, 111604, 1:100). CD45^+^CD11b^+^F4/80^+^ macrophages were sorted using a CytoFLEX SRT cell sorter (Beckman Coulter). Sorted cells were collected in cold FACS buffer, and post-sort purity routinely exceeded 95%.

**Culture and immunofluorescence staining of sorted macrophages:** Sorted CD45^+^CD11b^+^F4/80^+^ macrophages were seeded onto poly-L-lysine-coated glass coverslips in 24-well plates and cultured in medium supplemented with 10% FBS and 1% penicillin-streptomycin at 37°C in 5% CO_2_ for cell attachment. For immunofluorescence staining, adherent cells on coverslips were washed with PBS, fixed with 4% paraformaldehyde at room temperature for 10 min, and washed three times with PBS for 5 min each wash. Cells were then blocked with 5% BSA at room temperature for 1 hour and incubated overnight at 4°C with the following primary antibodies: CCR2 (1:100, MAB55382-100, Bio-Techne) and Ly6C (1:100, ab317272, Abcam). The next day, coverslips were washed three times with PBS for 5 minutes each and incubated with the appropriate fluorophore-conjugated secondary antibodies (1:400 in 5% BSA) at room temperature for 1 hour in the dark. After final washes, coverslips were mounted in DAPI Fluoromount-G® mounting medium for nuclear counterstaining. Images were acquired using a fluorescence microscope and processed with ImageJ software.

**Bone marrow derived macrophages (BMDMs) isolation and culture：**Macrophages were derived from bone marrow precursors as previously described^3,8^. Bone marrow cells (1 x 10^7^) were seeded in 10 cm cell culture dishes containing 6 mL of DMEM supplemented with 30% L929 cell-conditioned medium and 20% FBS. On days 3 or 4, an additional 3 mL of macrophage differentiation medium was added. On day 7, macrophages were centrifuged and harvested with ice-cold Ca^2+^ and Mg^2+^-free PBS. The harvested cells were confirmed to be macrophages using flow cytometry (FACS), with CD68 as a pan-macrophage marker.

**Peritoneal macrophages (PMs) isolation and culture:** Three days before the experiment, mice were i.p. injected with 1 ml of 6% starch solution to elicit peritoneal macrophages. Mice were euthanized and 10 ml of ice cold PBS was injected into the peritoneal cavity. Gently rub the abdomen for 1 minute, and the cell-containing PBS was collected into a 50 ml centrifuge tube. This washing process was repeated three times. The collected PBS suspension was centrifuged at 800 rpm for 5 minutes, and the cells were resuspended in high-glucose DMEM containing 10% FBS. The cells were counted and seeded into a cell culture incubator at 37°C. After 2 hours of adhesion, the medium was refreshed to obtain primary peritoneal macrophages.

**Primary neonatal rat ventricular myocytes (NRVMs) isolation and culture:** NRVMs were isolated and cultured as previously described^5^. In brief, hearts from 1- or 2‐day‐old Sprague Dawley rats were harvested. Cardiac ventricles were digested with 0.114% collagenase II (Biosharp, Shanghai, China) and 0.036% trypsin (Solarbio, Beijing, China) at 37°C. The cells were pre‐plated for 90 minutes to remove non‐cardiomyocytes. Cardiomyocytes were cultured in DMEM supplemented with 10% FBS and 0.1 mmol/L bromodeoxyuridine (BrdU, Sigma, St.Louis, MO, USA) to inhibit the proliferation of non‐cardiomyocytes for 24h.

***In vitro* BMDMs hypoxia and reoxygenation (HR) model：**To simulate BMDM HR model *in vitro*, BMDMs pretreated with or without inhibitors were replaced with serum-free DMEM, and then the BMDMs were placed in a hypoxic chamber at 37°C infused with a gas mixture containing 1% oxygen and 5% carbon dioxide for 12 hours, after that, the cells were placed in aerobic culture incubators for 12 hours. Collected the medium as the macrophages-conditioned medium for subsequent experiments. The following inhibitors used in this study were purchased from MedChemexpress‌: DFO (PHD inhibitor, HY-B0988), Chetomin (Hif-1α inhibitor, HY-107553), NF-κB-IN-1 (NF-κB inhibitor, HY-138537).

***In vitro* BMDMs H_2_O_2_ treatment model：**To mimic excessive ROS exposure during IR injury *in vitro*, BMDMs were treated with 150 μM H_2_O_2_ for 8 hours. And then the culture medium was collected, centrifuged at 1000 rpm for 5 minutes as the macrophage-conditioned medium.

**siRNA electroporation in BMDMs:** BMDMs were harvested and resuspended in electroporation buffer (Opti-MEM I) at a concentration of 1 x 10⁶ cells per 100 μL. A mixture of 1-2 μg BCAT1 siRNA or scrambled control siRNA was added to the cell suspension. Electroporation was performed using a 2-mm gap cuvette in a Bio-Rad Gene Pulser Xcell system (300 V, 200 μF, 200 Ω). After electroporation, cells were gently resuspended and plated in prewarmed culture medium containing 20 ng/mL M-CSF to support macrophage survival and differentiation. Cells were incubated at 37°C in 5% CO_2_ for 24-48 hours to allow gene silencing. Knockdown efficiency was assessed at the mRNA level by qPCR.

***In vitro* ischemia-reperfusion injury model:** To simulate myocardial ischemia-reperfusion injury *in vitro*, the culture medium for NRVMs was replaced with either from macrophages conditioned medium or DMEM containing 10% FBS. After 24 hours, cells underwent hypoxia-reoxygenation treatment. During the hypoxia phase, the culture medium was replaced with a modified Esumi ischemic buffer (117 mM sodium chloride, 12 mM potassium chloride, 0.49 mM magnesium chloride, 0.9 mM calcium chloride, 5.6 mM 2-deoxy-glucose, 4 mM HEPES, 20 mM sodium lactate, pH 6.2) to simulate a hypoxic microenvironment with elevated potassium ions and low pH. Cells were placed in a hypoxic chamber at 37°C infused with a gas mixture containing 0.1% oxygen and 5% carbon dioxide. Control cells were cultured in regular serum-free, high-glucose DMEM, and cells were incubated under standard conditions at 37°C during the reperfusion phase.

**CCK-8 assay:** BMDMs were seeded in 96-well plates at a density of 1 x 10^5^ cells per well in 200 µl of culture medium. At each measurement, the medium was replaced with 90 µl fresh medium and 10 µl Cell Counting Kit-8 reagent (DOJINDO), followed by incubation for 1 hour at 37 °C in the dark. Absorbance was then measured at 450 nm using a microplate reader.

**Metabolite supplementation**: The working concentrations and exposure durations of the cell-permeable metabolites dimethyl α-ketoglutarate (DM-αKG, HY-44134, MedChemExpress LLC) and dimethyl succinate (DM-succ, HY-Y0808, MedChemExpress LLC) were determined in dose- and time-response experiments (Supplementary Fig. 6f-i) as the highest concentrations and longest exposure durations that did not affect BMDM viability. Based on these results, 1 mM DM-αKG and 2.5 mM DM-succ were applied for 24 hours in all subsequent experiments.

**ELISA assay:** Blood samples were allowed to clot for 2 hours at room temperature and centrifuged at 12,000 rpm for 15 minutes at 4 °C. The culture medium from BMDMs was centrifuged to remove cell debris. Levels of TNFα, IL-6, and IL-10 in mouse serum and BMDMs culture medium were measured using ELISA kits (CLOUD‐CLONE CORP., CCC, USA) according to the manufacturer’s instructions. Concentrations were calculated using standard curves generated with CurveExert 1.4.

**Measurement of reactive oxygen species (ROS):** ROS in frozen sections of mouse heart tissue and macrophages was assessed using dihydroethidium (DHE) (YEASEN, Shanghai) following the manufacturer’s instructions. Intracellular ROS levels were analyzed using the fluorescent probe CM-H2DCFDA (Sigma, D6883) by Flow cytometry. Mitochondrial ROS were measured using MitoSOX™ Red (Invitrogen, M36008) according to the manufacturer’s protocol.

**ROS clearance:** MitoTEMPO (ENZO Life Science, ALX-430-150) was dissolved in DMSO to prepare a 10 mM stock solution. For *in vitro* experiments, BMDMs were pretreated with 10 μM MitoTEMPO for 24 hours before hypoxia-reoxygenation treatment.

**Oxidative DNA damage assay:** Oxidative DNA damage was assessed using the 8-hydroxy-2’-deoxyguanosine (8-OHdG) ELISA kit from Abcam (ab285254), following the manufacturer's protocol.

**Seahorse OCR and ECAR measurement:** BMDMs were seeded into XF96 cell culture microplates (Agilent) at a density of 5 × 10^5^ cells/mL. The oxygen consumption rate (OCR) and extracellular acidification rate (ECAR) were measured using the Seahorse XF96 Analyzer (Agilent) following the manufacturer's instructions. For the ECAR assay, Glycolysis was calculated as: Glycolysis = Maximum ECAR before oligomycin injection - Last ECAR before glucose injection. Glycolytic capacity was calculated as: Glycolytic capacity = Maximum ECAR after oligomycin injection - Last ECAR before glucose injection. For the OCR assay, basal respiration was calculated as: Basal respiration = Last OCR measurement before the first injection - Non-mitochondrial respiration rate. Maximal respiration was calculated as: Maximal respiration = Maximum OCR after FCCP injection - Non-mitochondrial respiration rate. ATP-linked OCR (ATP production) was calculated as: ATP-linked OCR = Last OCR before oligomycin injection - Minimum OCR after oligomycin injection.

**NADH/NAD^+^, NADPH/NADP^+^ and GSH/GSSG assays:** The levels of NADH/NAD^+^, NADPH/NADP^+^ and GSH/GSSG were measured using the NADH/NAD^+^ assay kit (ab65348), NADPH/NADP^+^ assay kit (ab65349), and GSH/GSSG detection kit (Beyotime, S0053), respectively, following the manufacturer’s instructions.

**Fe^2+^ assay:** The levels of Fe^2+^ were measured using the Iron Assay Kit (Dojindo, I291) according to the manufacturer’s instructions.

**^13^C tracing metabolic flux analysis:** For ^13^C tracing experiments, BMDMs were cultured in medium supplemented with 2 mM [U-^13^C_5_]-L-glutamine (CIM-1822-H-0.25, Cambridge Isotope Laboratories, USA) for 48 hours. Control wells received non-isotopically labeled L-glutamine. Isotope labeling data were acquired using a Shimadzu LC system coupled with a Triple TOF QTOF 6600+ mass spectrometer (AB Sciex, Woodlands Central Industrial Estate, Singapore). Chromatographic separation was performed on an IHILIC-(P) Classic column (5 μm, 150 mm × 2.1 mm, 200 Å, Sweden). Mobile phase A consisted of 20 mmol/L ammonium acetate, 0.1% ammonium hydroxide, and 2.5 μmol/L methylene diphosphate in water/ACN (95:5), and mobile phase B was ACN. The gradient was as follows: 0 min, 85% B; 2 min, 85% B; 7 min, 65% B; 12 min, 35% B; 12.1 min, 20% B; 15.9 min, 20% B; 16 min, 85% B; 23 min, 85% B. The flow rate was maintained at 0.2 mL/min, with an injection volume of 5 μL and a total run time of 23 min. ESI parameters were configured as follows: GS1, 60; GS2, 60; CUR, 35; temperature, 500; ISVF, −4500 in negative mode. For each metabolite, the areas of ^12^C and various ^13^C isotopologue peaks were integrated using El-MAVEN software (version 0.12.1). Natural isotope abundance correction was performed using the R package AccuCor. The fractional abundance of each isotopologue was calculated as the area of that isotopologue divided by the total area of all isotopologues.

**Immunofluorescent staining:** Five-μm-thick cryosections were prepared from frozen hearts embedded in optimal cutting temperature compound (OCT). For cell staining, bone marrow-derived macrophages (BMDMs) cultured on glass coverslips were used. Both sections and coverslips were washed with PBS and fixed with 4% paraformaldehyde at room temperature for 10 minutes. Slides were washed three times with PBS for 5 minutes each time and blocked with 5% BSA at room temperature for 1 hour. Sections were incubated overnight at 4℃ with the following primary antibodies: CD68 (1:250, MCA1957GA, Bio-Rad), GLUD1 (1:200, ab166618, Abcam), p65 (1:200, 8242, CST), CD206 (1:500, AF2535, R&D), iNOS (1:100, ab15323, Abcam), and eGFP (1:100, CAB4211, Thermo Fisher). The next day, slides were washed three times with PBS for 5 minutes each time and incubated with secondary antibodies (1:400 in 5% BSA) at room temperature for 1 hour. Finally, slides were mounted with DAPI Fluoromount-G® and imaged using a fluorescence microscope. Images were processed using ImageJ software.

**TUNEL staining:** Cryosections and cultured cardiomyocytes were stained using the terminal deoxynucleotidyl transferase‐mediated dUTP nick‐end labeling (TUNEL) method with an In Situ Cell Death detection kit (Roche, Basel, Switzerland) to detect apoptosis. Cryosections were fixed with 4% paraformaldehyde and permeabilized with 0.1% Triton X‐100. Cell nuclei were stained with DAPI.

**Extraction of** **nuclear proteins**: Nuclear and cytoplasmic proteins were extracted using the Cell Nuclear and Cytoplasmic Protein Extraction Kit (Bi Yun Tian Biotechnology Co., Ltd., Shanghai, China) according to the manufacturer’s instructions.

**Immunoblotting:** Cells and tissues were lysed in radioimmunoprecipitation assay (RIPA) buffer supplemented with protease and phosphatase inhibitors, followed by homogenization. A protein load (from 10 to 20 μg) was separated by SDS‐PAGE and transferred to polyvinylidene difluoride (PVDF) membranes (Millipore, Billerica, MA, USA). After blocking, membranes were incubated overnight at 4℃ with primary antibodies, followed by incubation with HRP-conjugated secondary antibodies for 1 hour at room temperature. Proteins were detected using a Bio-Rad ChemiDoc MP Imaging system with enhanced chemiluminescence. The following antibodies were used: GLUD1 (1:1000, ab166618, Abcam), HIF-1α (1:1000, NB100-105, Novus), IκBα (1:1000, 4812, CST), P-p65 (1:1000, 3033, CST), p65 (1:1000, 8242, CST), CD206 (1:1000, ab64693, Abcam), iNOS (1:1000, ab15323, Abcam), Lamin A/C (1:1000, 4777, CST), cleaved caspase-3 (1:1000, 9661, CST), caspase-3 (1:1000, 19677-1-AP, Proteintech), Vinculin (1:1000, 42H89L44, Thermo Fisher), β-actin (1:5000, HRP-60008, Proteintech).

**Single-cell RNA sequencing analysis:** The mouse scRNA-seq dataset (GSE247139) was obtained from a public repository. Analysis and visualization were performed using the R package Seurat v4.3.1. Cells expressing fewer than 200 or more than 4000 genes, as well as those with mitochondrial gene content exceeding 20%, were excluded. Data were normalized using the “NormalizeData” function. The top 2000 variable genes were selected using “FindVariableFeatures” and scaled with “ScaleData” for principal component analysis (PCA). The datasets were then integrated using “Harmony”.

Cell clusters were manually annotated according to canonical marker genes. The following cell types were identified in the dataset: macrophages (*Cd68*, *Fcgr1*, *Adgre1*), endothelial cells (*Vwf*, *Pecam1*, *Cdh5*), fibroblasts (*Gsn*, *Pdgfra*, *Dcn*), T cells (*Cd4*, *Cd3e*), NK cells (*Klrb1c*, *Ncr1*), B cells (*Cd19*, *Cd79a*, *Cd79b*), pericytes (*Cspg4*, *Vtn*), smooth muscle cells (*Acta2*, *Myh11*), neutrophils (*S100a8*, *S100a9*), cardiomyocytes (*Tnnt2*, *Myl2*, *Tnnc1*), Schwann cells (*Sox10*, *Plp1*), epithelial cells (*Krt19*, *Upk3b*, *Msln*). Macrophages were further subdivided into Ly6C^+^ monocytes, BLT1^+^ macrophages, S100a9^+^ macrophages, ISG^+^ macrophages, MHC-II^+^ macrophages, Mt^hi^ macrophages and Lyve1^+^ macrophages.

The expression levels of *Glud1* and the inflammation score were calculated using “AddModuleScore”. To investigate the differentiation trajectory of monocytes recruited to the heart after IR injury, pseudotime analysis was performed using Monocle2. Resident Lyve1⁺ macrophages were excluded to minimize confounding effects from tissue-resident populations. The remaining monocytes/macrophages were used to construct the differentiation trajectory. Monocle2 was applied with default parameters unless otherwise specified, and the branching tree was inferred based on differentially expressed genes across pseudotime.

**RNA sequencing and gene expression analysis**: RNA sequencing (RNA-seq) was conducted by Shanghai Majorbio Biopharm Technology Co., Ltd. (Shanghai, China). Total RNA was extracted from peripheral blood monocytes of acute myocardial infarction (AMI) patients and healthy donors using TRIzol reagent (Sigma-Aldrich) and treated with RNase-free DNase I. RNA integrity was assessed using an Agilent Bioanalyzer. Complementary DNA (cDNA) was synthesized using the ReverAid^TM^ First Strand cDNA Synthesis Kit (Thermo Fisher Scientific). RNA libraries were prepared and sequenced on HiSeq XTen sequencers (Illumina, San Diego, CA). Quality control of the raw reads was performed using the FastQC tool. The clean sequenced reads were aligned to the reference genome using HISAT2. Differential gene expression was computed and determined using the DESeq2 package. Significantly differentially expressed genes were defined as a false discovery rate (FDR) < 0.05 with foldchange >1. Gene ontology and KEGG analysis were carried out using the clusterProfiler R package with FDR < 0.05.

**Statistical analysis:** Statistical analyses were performed using GraphPad Prism version 9.0 (GraphPad Software). No data were excluded from the analyses, and no transformation was performed before statistical testing. Western blot quantifications were normalized to the loading controls, and qPCR data were normalized to internal reference genes. Data in Figures 4c,f, 5a-b, 6m-o and 7d, as well as Supplementary Fig. 6f-i and n-o, are presented as fold change relative to the mean of the corresponding control group; each replicate was normalized individually. Data distribution was assessed using the Shapiro-Wilk test. For comparisons between two groups, an unpaired two-tailed Student’s *t*-test was used for normally distributed data, and the Mann-Whitney *U* test was used for non-normally distributed data. For comparisons among more than two groups, one-way ANOVA was used when one factor was varied, and two-way ANOVA was used when two factors were varied, followed by Tukey's or Sidak's post hoc tests, as appropriate. Data are presented as mean ± standard error of the mean (SEM). Mean values were calculated from replicates, and the numbers of independent experiments and biological samples are indicated in each figure legend. A *P* value < 0.05 was considered statistically significant.

**References:**

1	Carobbio, S., Frigerio, F., Rubi, B. *et al.* Deletion of glutamate dehydrogenase in beta-cells abolishes part of the insulin secretory response not required for glucose homeostasis. *The Journal of biological chemistry* **284**, 921-929 (2009).

2	Li, J., Chen, K., Zhu, L. & Pollard, J. W. Conditional deletion of the colony stimulating factor-1 receptor (c-fms proto-oncogene) in mice. *Genesis* **44**, 328-335 (2006).

3	Shang, M., Cappellesso, F., Amorim, R. *et al.* Macrophage-derived glutamine boosts satellite cells and muscle regeneration. *Nature* **587**, 626-631 (2020).

4	Lv, Q., Li, D., Zhao, L. *et al.* Proline metabolic reprogramming modulates cardiac remodeling induced by pressure overload in the heart. *Sci Adv* **10**, eadl3549 (2024).

5	Lin, J., Li, Q., Jin, T. *et al.* Cardiomyocyte IL-1R2 protects heart from ischemia/reperfusion injury by attenuating IL-17RA-mediated cardiomyocyte apoptosis. *Cell Death Dis* **13**, 90 (2022).

6	Sun, S., Lai, C., Huang, C. *et al.* Exercise-induced histone lactylation in monocyte-derived macrophages restores cardiac immune homeostasis and function in sepsis-induced cardiomyopathy. *Nat Commun* **17**, 756 (2025).

7	Wang, N., Wang, W., Wang, X. *et al.* Histone Lactylation Boosts Reparative Gene Activation Post-Myocardial Infarction. *Circ Res* **131**, 893-908 (2022).

8	Wenes, M., Shang, M., Di Matteo, M. *et al.* Macrophage Metabolism Controls Tumor Blood Vessel Morphogenesis and Metastasis. *Cell Metab* **24**, 701-715 (2016).
